# Amine Basicity of Quinoline ATP Synthase Inhibitors Drives Antibacterial Activity against *Pseudomonas aeruginosa*

**DOI:** 10.1021/acsmedchemlett.3c00480

**Published:** 2023-12-26

**Authors:** Katie T. Ward, Alexander P. L. Williams, Courtney A. Blair, Ananya M. Chatterjee, Abirami Karthikeyan, Addison S. Roper, Casey N. Kellogg, P. Ryan Steed, Amanda L. Wolfe

**Affiliations:** Department of Chemistry and Biochemistry, University of North Carolina Asheville, One University Heights, Asheville, North Carolina 28804, United States

**Keywords:** ATP synthase, Bioenergetics, Antibiotics, Antibiotic resistance, *Pseudomonas aeruginosa*, Structure−activity relationship analysis

## Abstract

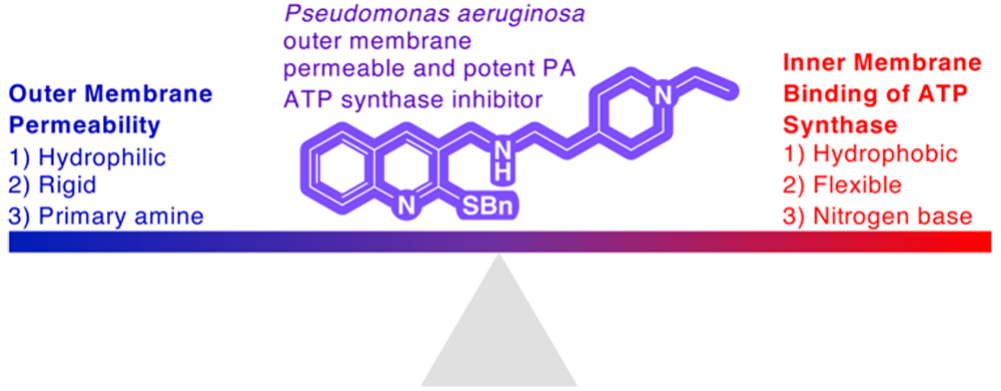

*Pseudomonas aeruginosa* (PA), a Gram-negative pathogen, is a common cause of nosocomial infections, especially in immunocompromised and cystic fibrosis patients. PA is intrinsically resistant to many currently prescribed antibiotics due to its tightly packed, anionic lipopolysaccharide outer membrane, efflux pumps, and ability to form biofilms. PA can acquire additional resistance through mutation and horizontal gene transfer. PA ATP synthase is an attractive target for antibiotic development because it is essential for cell survival even under fermentation conditions. Previously, we developed two lead quinoline compounds that were capable of selectively inhibiting PA ATP synthase and acting as antibacterial agents against multidrug-resistant PA. Herein we conduct a structure–activity relationship analysis of the lead compounds through the synthesis and evaluation of 18 quinoline derivatives. These compounds function as new antibacterial agents while providing insight into the balance of physical properties needed to promote cellular entry while maintaining PA ATP synthase inhibition.

*Pseudomonas aeruginosa* (PA), a Gram-negative, biofilm-forming bacterium, is one of the leading causes of nosocomial (or healthcare-acquired) pneumonia, surgical site infections, bloodstream infections, and catheter-associated urinary tract infections. Additionally, a recent international study found PA to be the source of approximately 23% of patient infections in intensive-care units.^1−3^ Cystic fibrosis (CF) patients are particularly susceptible to PA infections, which are the leading cause of death in this population, making treatment of PA infection a standard of care for CF.^2^ Despite the prevalence of nosocomial PA infections, treatment options are limited, with the standard treatments being β-lactam and/or aminoglycoside antibiotics. However, multidrug-resistant (MDR) PA strains are on the rise, with the Centers for Disease Control (CDC) reporting that 9% of all PA isolates in the United States in 2017 were MDR. Data from the CDC National Healthcare Safety Network reports that 16.8% of ICU patients and 39% of long-term care patients with ventilator-associated PA infections were resistant to three or more antibiotics.^1,4^ PA utilizes both intrinsic (efflux pumps, low outer membrane (OM) permeability, biofilm formation, etc.) and acquired (via mutation or horizontal gene transfer) resistance mechanisms to overcome antibiotic action; therefore, the development of new antibiotics that treat MDRPA infections is desperately needed.^5^

Since the development of bedaquiline (BDQ), an antitubercular antibiotic, by Johnson and Johnson in the early 2000s,^6^ bacterial ATP synthase has been an attractive target for antibiotic development due to the role of ATP synthase in bioenergetics and pH homeostasis.^5,7,8^ Additionally, unlike other bacteria, PA relies on ATP synthase for ATP production even during anaerobic growth, making it an even more attractive target for antibiotic and antibiotic adjuvant development.^7,8^ ATP synthase (Figure 1A) is a membrane-embedded protein complex that harnesses energy from rotation of its multisubunit F_0_ domain to synthesize ATP in the multisubunit F_1_ domain. Rotation of F_0_ is driven by protons moving along their electrochemical gradient.^9^ The bacterial F_0_ motor is composed of a rotor of 10–15 copies of the *c* subunit adjacent to subunit *a* and a dimer of *b* subunits that form the stator. Each *c* subunit contains a proton binding site (Asp60 in PA) in the middle of the membrane that is accessed by two aqueous half channels in subunit *a*.^8−11^

**Figure 1:**
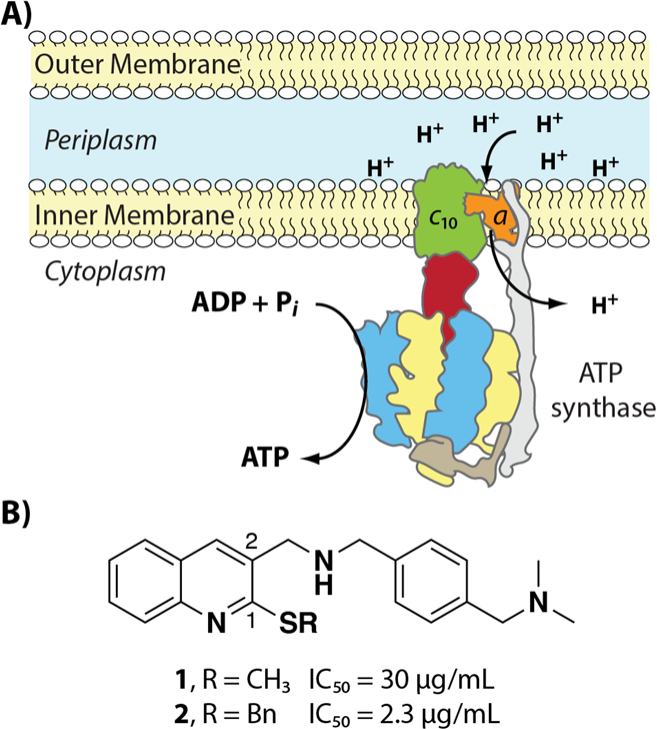
(A) Cartoon showing the overall structure, function, and location of ATP synthase in the inner membrane of PA. (B) Structures of lead PA ATP synthase inhibitors **1** and **2** and previously determined IC_50_ values for inhibition of PA ATP synthase activity.^11^

PA ATP synthase is embedded in the inner membrane (IM) of PA, which is a phospholipid bilayer. A major challenge in the development of small-molecule ATP synthase inhibitors is that the molecules must have physical properties that allow them not only to enter the hydrophobic IM to reach the binding site on the *c* subunit of ATP synthase but also to traverse the asymmetric, polyanionic lipopolysaccharide OM that encapsulates the cell and avoid expulsion by promiscuous efflux pumps. Recently, we developed a series of quinoline-based inhibitors of PA ATP synthase that mimic BDQ by binding to the proton binding site of the *c* subunit of PA ATP synthase.^10,11^ Of those, only compounds **1** and **2** (Figure 1B), which have a 1-(4-(aminomethyl)phenyl)-*N*,*N*-dimethylmethanamine off of the quinoline C2 and either a methyl sulfide or benzyl sulfide off of the quinoline C1, respectively, were capable of both inhibiting PA ATP synthase and acting as antibiotics against clinical isolates of MDRPA by successfully crossing the OM.^11^ Herein we report a structure–activity relationship (SAR) study of 18 synthetic quinoline analogs derived from compounds **1** and **2**, which resulted in more potent PA ATP synthase inhibitors and a better understanding of the physical properties required to promote antibacterial activity.

To explore the SAR profile of compounds **1** and **2**, a series of C2 amine derivatives (**5**–**22**) were synthesized via a one-pot, two-step reductive amination reaction starting from either 2-(methylthio)quinoline-3-carbaldehyde (**3**) or 2-(benzylthio)quinoline-3-carbaldehyde (**4**) in moderate yields (Scheme 1). This series of amines was chosen to probe the effects of the size, rigidity, aromaticity, and lipophilicity of C2 on both ATP synthesis inhibition and antibacterial activity against PA.

**Scheme 1:**
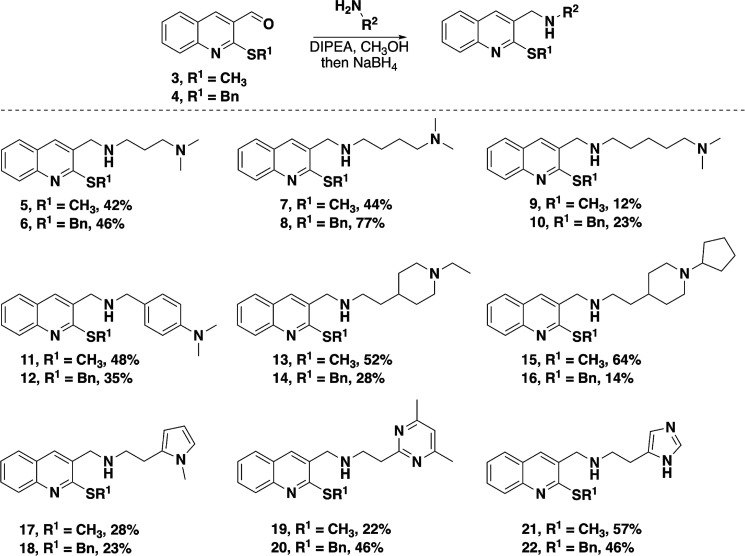
Synthesis of Compounds **5**–**22** via Reductive Amination Chemistry

Compounds **5**–**22** were evaluated for their ability to inhibit *in vitro* NADH-driven PA ATP synthesis activity in DK8/pASH20 inverted membrane vesicles, which were prepared from expression of PA ATP synthase in *Escherichia coli* (EC) DK8 (a K-12 strain lacking an endogenously encoded ATP synthase) as previously described, using an end-point luciferin/luciferase assay at increasing concentrations of each compound.^11^ When compared to the BDQ binding site on the *c* subunit of *Mycobacterium tuberculosis* ATP synthase, the analogous site on the *c* subunit of PA ATP synthase is less sterically congested. Therefore, compound **2** showed greater PA ATP synthase activity (IC_50_ = 2.3 μg/mL) compared to compound **1** (IC_50_ = 30 μg/mL) due to the larger C1 benzyl sulfide on **2** compared to the C2 methyl sulfide on **1**.^11^ This trend was generally observed/confirmed in this series, as seen in Figure 2, where compounds with a benzyl sulfide at C1 showed greater inhibition of ATP synthase activity compared with compounds with a methyl sulfide at C1 with the same C2 substitution. Only compounds **17** (SCH_3_) and **18** (SBn), with 2-(1-methyl-1*H*-pyrrol-2-yl)ethanamine substituted at C2, showed similar PA ATP synthase inhibition. Compound **12**, with a C1 SBn and C2 dimethylaniline, showed potent PA ATP synthase inhibition at low concentrations (<4 μg/mL); however, due to poor solubility in the assay medium at concentrations >16 μg/mL, an IC_50_ could not be determined. Even at the highest concentrations tested, many of the compounds with a methyl sulfide at C1 showed incomplete inhibition of ATP synthesis. Absolute IC_50_ values were determined for these compounds based on the assumption that binding completely inhibits activity, consistent with the mechanism of BDQ,^12^ and partial inhibition indicates partial occupancy of the inhibitor binding site.

**Figure 2:**
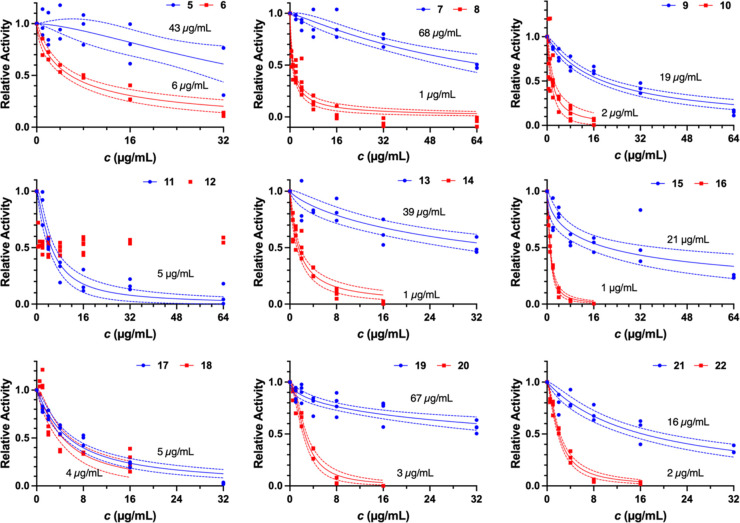
Inhibition of PA ATP synthase activity. Inverted membrane vesicles from EC DK8 cells expressing PA ATP synthase were tested for NADH-driven ATP synthesis activity in the presence of each compound as described in Experimental Procedures in the Supporting Information. Relative activities (normalized to uninhibited ATP synthesis activity) in the presence of *S*-methyl (blue circles) or *S*-benzyl (red squares) compounds were fit using a dose–response model (solid curves). Confidence bands (≥95%) are plotted for each fit as dashed lines, and resulting absolute IC_50_ values are reported for each fit.

Of the new C1 benzyl sulfide compounds, compounds **8** (C2 = *N*,*N*-dimethyl-1,4-butanediamine), **14** (C2 = 2-(1-ethylpiperidin-4-yl)ethanamine), and **16** (C2 = 2-(1-cyclopentylpiperidin-4-yl)ethanamine) showed the greatest PA ATP synthase inhibition with IC_50_ = 1 μg/mL. Location of the C2 nitrogen on the side chain (i.e., its distance is closer or further from the quinoline in space) did affect PA ATP synthase inhibition, with compounds **8**, **14**, and **16** having the nitrogen a similar distance from the quinoline in space (Figure 3). We hypothesize that the nitrogen likely interacts with Asp60 in the *c* subunit binding site, but further studies are needed to confirm this since the structure of PA ATP synthase has not been elucidated. The next most potent PA ATP synthase inhibitors in the series were compounds **2**, **10**, and **22**, with IC_50_ values of approximately 2 μg/mL. Compounds **2** and **10** have a longer distance between C2 and the dimethylamine nitrogen compared to compounds **8**, **14**, and **16**, whereas compound **22** has a slightly shorter distance between the C2 and imidazole nitrogens. Compound **6**, which shortens the C2 side chain by one carbon compared to **8**, had slightly decreased PA ATP synthase inhibition (IC_50_ = 6 μg/mL). Compounds **18** (IC_50_ = 5 μg/mL) and **20** (IC_50_ = 3 μg/mL) also have their C2 nitrogen functionality closer to the quinoline core and showed less PA ATP synthase inhibition activity; however, steric bulk around the terminal nitrogen in the C2 group seems to improve PA ATP synthase inhibition when comparing these to compound **6**. Finally, within the C1 benzyl sulfide series, larger, hydrophobic functional groups at the end of the C2 group are well-tolerated, as seen with compounds **14**, **16**, and **20**.

**Figure 3:**
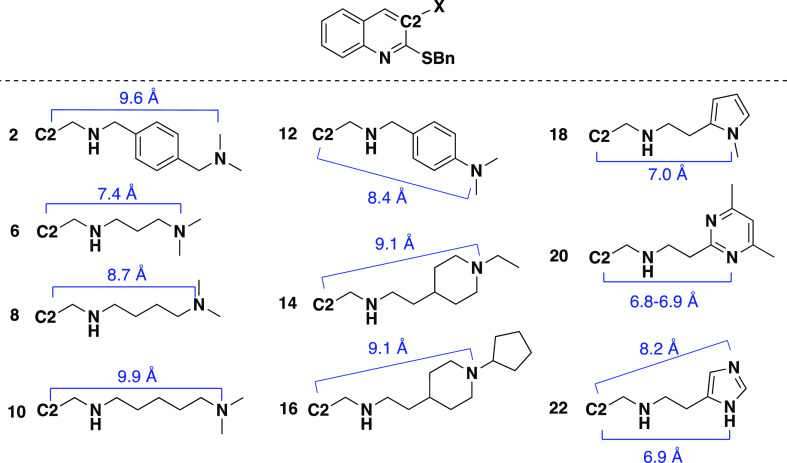
Side chain lengths calculated in Chem3D after MM2 energy minimization.

As a control for off-target electron transport chain (ETC) inhibition in both the PA ATP synthase assay and the antibacterial assay against PA strains, compounds **5**–**22** were evaluated for their ability to inhibit the EC and PA ETCs (Tables 1 and S1). The assay for PA ATP synthase activity in EC DK8/pASH20 membrane vesicles requires a functional EC ETC, so potent inhibition of the EC ETC by any compound would interfere with the determination of its IC_50_ for PA ATP synthase. Only compound **22** (EC ETC IC_50_ = 14 μg/mL) inhibited EC ETC within 10-fold of the measured PA ATP synthase inhibition activity in DK8/pASH20 membrane vesicles (Table S1). Previously, compound **1** was shown to inhibit PA ETC with IC_50_ = 29 μg/mL, which is equal to its PA ATP synthase IC_50_ (30 μg/mL) in these vesicles.^11^ Furthermore, potent inhibition of PA ETC could be an alternative mechanism of action for the antibacterial activity of the current series. Similar to the observed EC ETC inhibition, only compounds **6** and **22** had PA ETC within 10-fold of their PA ATP synthase IC_50_ concentrations. This indicates that the antibacterial activity (described in Table 2) of compounds **2**, **6**, **8**, **10**, **14**, and **16** is due to PA ATP synthase inhibition and not PA ETC inhibition.

**Table 1:** Inhibition of PA Electron Transport Chain of **1**, **2**, **6**, **8**, **10**, **14**, **16**, and **22**

compound	PA ETC IC_50_ (μg/mL) [fold difference]^a^
**1**(11)	29 [1]
**2**(11)	27 [12]
**6**	44 [7]
**8**	26 [26]
**10**	57 [28]
**14**	26 [26]
**16**	14 [14]
**22**	15 [7]

a Fold difference = [PA ETC IC_50_]/[PA ATP synthase IC_50_]

**Table 2:** Antibacterial Activities of Compounds **1**, **2**, and **5**–**22**

	MIC (μg/mL)^a^^,^^b^				
compound	**PA 9027**	**PΔ6**	**BAA 2108**	**BAA 2109**	**BAA 2110**
**1**(11)	>256	64–128	128–256	256	>256
**2**(11)	>128	16	64	>128	>128
**5**	>128	>128	>128	>128	>128
**6**	>128	16	64	64	32
**7**	>128	>128	>128	>128	>128
**8**	>128	16	64	64	32
**9**	>128	128	>128	>128	>128
**10**	>128	64	64	128	64
**11**	>128	128	>128	>128	>128
**12**	>128	>128	>128	>128	>128
**13**	>128	128	>128	>128	>128
**14**	>128	16	32	64	16
**15**	>128	128	>128	>128	>128
**16**	>128	8	32	64	>128
**17**	>128	128	>128	>128	>128
**18**	>128	>128	>128	>128	>128
**19**	>128	128	>128	>128	>128
**20**	>128	>128	>128	>128	>128
**21**	>128	128	>128	>128	>128
**22**	>128	16	32	64	>128
**chloramphenicol**	128	4	32	128	128
**gentamicin**	2–4	>4	16	2	4–8

a MIC = minimum inhibitory concentration of >85% reduction in pathogen growth with compound compared to pathogen alone (DMSO only) at OD 590 nm (no visible growth).

b *n* = 3.

Translating PA ATP synthase inhibition into whole-cell antibacterial activity against antibiotic-resistant PA strains is challenging because, as discussed above, ATP synthase is embedded in the hydrophobic inner membrane. To access their binding target, molecules must traverse the more hydrophilic and anionic outer membrane, creating a need to strike a balance between hydrophobicity and hydrophilicity. To determine this balance, compounds **5**–**22** were evaluated for antibacterial activity against a nonvirulent, biofilm-forming strain of PA (designated PA 9027),^13^ an efflux knockout (KO) strain of PA (designated PΔ6),^14^ and three MDRPA clinical isolates (ATCC BAA 2108, BAA 2109, and BAA 2110) from cystic fibrosis patients that are broadly resistant to penicillin and cephalosporin antibiotics, tigecycline, and nitrofurantoin and susceptible to quinolone and aminoglycoside antibiotics (Table 2). Generally, as seen with the ATP synthase inhibition, C1 benzyl sulfides were more active than the analogous C1 methyl sulfides, of which only **9**, **11**, **13**, **15**, and **19** displayed weak antibacterial activity against efflux KO strain PΔ6. None of the compounds evaluated could overcome biofilm formation against PA 9027. Compounds **6**, **8**, **10**, and **14** were broadly active against PΔ6 and the three MDRPA strains with MICs between 16 and 128 μg/mL. Compound **14** was the most potent of the series, with MICs against BAA 2108, 2109, and 2110 of 32, 64, and 16 μg/mL, respectively, and compound **16** had the lowest MIC (8 μg/mL) of the series against efflux KO strain PΔ6 but was inactive against BAA 2110 at the highest concentration tested. Compound **22** also displayed antibacterial activity against PΔ6, BAA 2108, and BAA 2109, but as stated, some of this activity is due to PA ETC inhibition, as was seen with compound **1** previously. C2 benzyl sulfides **12**, **18**, and **20** were inactive against all PA strains.

Recently, to aid in the discovery of Gram-negative antibiotics, rules of entry for Gram-negative bacteria, derived from evaluation of EC OM-penetrating drugs, have been established, which are (i) molecular weight <500 g/mol; (ii) cLogD_7.4_ between −2 and 0; (iii) ≤5 rotatable bonds; (iv) high polar surface area (average 165 Å); (v) low globularity; and (vi) presence of a 1° amine or guanidinium.^14−16^ While these rules do translate to other Gram-negative pathogens like *Acinetobacter baumannii*, PA has proven to be much more limited in chemical motifs that promote accumulation.^17^ Evaluation of the compounds in this series with regard to the entry rules would indicate that none in the series readily cross the OM of PA (Table S2). All compounds have molecular weights below 500 g/mol, but those with higher molecular weights show greater PA ATP synthase inhibitory and antibacterial activity in general (Figure 4). No trend between PA ATP synthase inhibitory or antibacterial activity and globularity can be defined, but all globularities are categorized as low according to the entry rules (Table S2). Unsurprisingly, the more flexible (>5 rotatable bonds) and hydrophobic (cLogP > 2) compounds showed the highest PA ATP synthase inhibitory activity since the IM is composed of hydrophobic fatty acids, but surprisingly, these also demonstrated the highest antibacterial activity against PA strains as well (Figure 4) despite the entry rules. Additionally, strong inhibition of ATP synthase alone (i.e., PA ATP synthase IC_50_ < 10 μg/mL) did not directly translate to PA antibacterial activity. For example, compounds **12**, **18**, and **20** showed no antibacterial activity despite having similar physical properties and ATP synthase inhibitor activity as **2**, **6**, **8**, **10**, **14**, **16**, and **22**.

**Figure 4:**
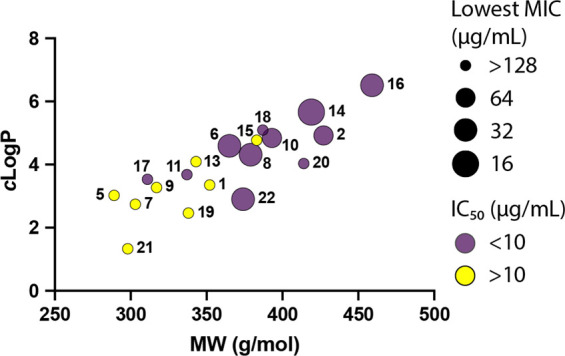
Comparison of molecular weight (MW) and hydrophobicity (cLogP) vs ATP synthase inhibition and antibiotic activity of compounds **1**, **2**, **5**–**11**, and **13**–**22**. Points representing each compound are sized by the lowest MIC against any of the MDRPA strains and colored by IC_50_ of ATP synthase inhibition.

All compounds in the series are nitrogen bases with the quinoline core, the benzylic secondary amine at C2, and an additional nitrogen functional group at the end of the C2 side chain. As can be seen in Table 3 and Figure 5, the approximate p*K*_a_ of the conjugate acid (NH^+^) of the C2 side chain varies across the series, with the tertiary piperidines (**13**–**16**) and tertiary dimethylamines (**1**, **2**, **5**–**10**) being the most basic and the *N*-methylpyrroles (**17** and **18**) and pyrimidines (**19** and **20**) being the least basic. The p*K*_a_ value of this position directly affects antibacterial activity. For potent PA ATP synthase inhibition to translate into potent antibacterial activity against PA strains, the p*K*_a_ of the conjugate acid of the C2 side chain needs to be above 6. For example, compounds **12**, **18**, and **20**, which are inactive against all tested strains of PA, with side-chain p*K*_a_ values <6, have similar PA ATP synthase IC_50_ concentrations (Figure 2) and physical properties (Figure 4 and Table S2) as compounds **6**, **10**, **14**, and **22**, which are active against multiple strains of PA, but with side chain p*K*_a_ values >6.

**Table 3:** C2 Side Chain Conjugate Acid p*K*_a_

compounds	C2 functional group	approximate p*K*_a_ (NH^+^)^a^
**1**, **2**, **5**–**10**	tertiary dimethylamine	∼10
**11**, **12**	dimethylaniline	∼5
**13**–**16**	tertiary piperidine	∼11
**17**, **18**	*N*-methylpyrrole	∼0.5
**19**, **20**	pyrimidine	∼1
**21**, **22**	imidazole	∼7

a Approximate p*K*_a_ values were taken from “Hans Reich’s Collection: Bordwell p*K*_a_ Table”.^19^

**Figure 5:**
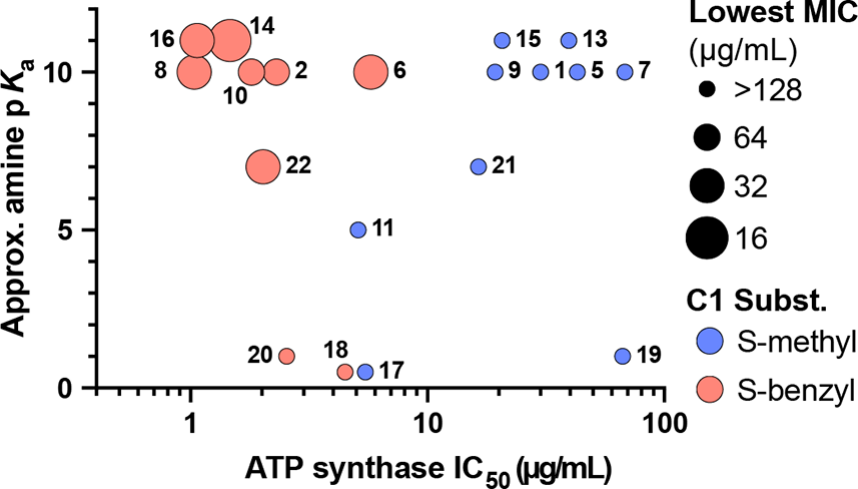
C2 amine basicity vs ATP synthase inhibition and antibacterial activity of compounds **1**, **2**, **5**–**11**, and **13**–**22**. Points representing each compound are sized by the lowest MIC against any of the MDRPA strains and colored by the C1 substituent.

Due to the promising antibacterial activity of lead compounds **1** and **2**, a series of 18 new quinoline analogs were synthesized via a one-pot, two-step reductive amination sequence at C2 starting from 2-(methylthio)quinoline-3-carbaldehyde (**3**) or 2-(benzylthio)quinoline-3-carbaldehyde (**4**). Once synthesized, each analogue was evaluated for PA ATP synthase inhibition activity, PA and EC electron transport chain inhibition activity, and antibacterial activity against MDRPA clinical isolates. As seen with compounds **1** and **2**, analogs with a benzyl sulfide at C1 of the quinoline generally showed greater inhibition of PA ATP synthase compared to compounds with the methyl sulfide at this position. PA ATP synthase inhibition was also weakly affected by the size, flexibility, and length of the side chain at C2 of the quinoline, with compounds **8**, **14**, and **16** showing the greatest activity. Of the most active PA ATP synthase inhibitors, only compounds **6** and **22** inhibited the PA ETC within 10-fold of their PA ATP synthase IC_50_ values, unlike lead compound **1**, which had equipotent PA ETC and PA ATP synthase inhibition activity. This indicates that this series is more selective for PA ATP synthase over PA ETC compared with the lead compounds.

C1 benzyl sulfides **6**, **8**, **10**, **14**, **16**, and **22** displayed the most potent antibacterial activity against the panel of PA strains examined, including three MDRPA clinical isolates and an efflux KO PA strain. Antibacterial activity of the most potent compounds was similar against the MDRPA isolates and the efflux KO PA strain, indicating that these molecules are not readily effluxed. However, none of the compounds evaluated was capable of overcoming biofilm formation of the PA 9027 laboratory strain. Lack of direct correlation between PA ATP synthase inhibition and antibacterial activity against PA indicated that OM penetration affects antibacterial activity. Evaluation of the physical properties of the series showed that these compounds do not follow the entry rules for Gram-negative bacteria. Both the antibiotic active (**6**, **8**, **10**, **14**, **16**, and **22**) and inactive (**12**, **18**, and **20**) analogs with similar PA ATP synthase inhibition activity were more flexible (rotatable bonds ≥8) and more hydrophobic (cLogP > 2.5) than preferred by the entry rules and did not contain a primary amine or guanidinium. Interestingly, the relative basicity of the nitrogen (NH^+^) side chain at C2 directly correlated with the antibacterial activity of analogs. The side chains of compounds **6**, **8**, **10**, **14**, **16**, and **22** have a nitrogen with a conjugate acid p*K*_a_ > 6, whereas those of compounds **12**, **18**, and **20** have a nitrogen with a conjugate acid p*K*_a_ < 6. While this trend needs to be further examined, this work indicates that the physical properties required for IM binding do not have to be incompatible with OM penetration in Gram-negative pathogens as has been seen previously,^18^ which will increase the number of biological targets available for antibiotic development in the future.

## Supporting Information Available

Experimental procedures and ^1^H and ^13^C NMR spectra for compounds **5**–**22**, control experiments for inhibition of *E. coli* DK8 pASH20 electron transport chain and PA electron transport chain (Table S1), and physical properties for compounds **1**, **2**, and **5**–**22** (Table S2) (PDF)

## Abbreviations

ATP: adenosine triphosphate
CF: cystic fibrosis
OM: outer membrane
IM: inner membrane
ETC: electron transport chain
PA: *Pseudomonas aeruginosa*
EC: *Escherichia coli*
MDR: multidrug-resistant
MIC: minimum inhibitory concentration
IC_50_: inhibitory concentration of 50%
SAR: structure–activity relationship
DMSO: dimethyl sulfoxide
DCM: dichloromethane
TSB: tryptic soy broth
Bn: benzyl
KO: knockout
