# Supplementary material for: Amine Basicity of Quinoline ATP Synthase Inhibitors Drives Antibacterial Activity against Pseudomonas aeruginosa
Source: ACS Med Chem Lett. 2023 Dec 26;15(1):149–55. doi: 10.1021/acsmedchemlett.3c00480 (PMC10789121; doi:10.1021/acsmedchemlett.3c00480)
Supplement: Supplementary file 1 — ml3c00480_si_001.pdf [file ml3c00480_si_001.pdf]

**Supporting Information: Amine Basicity of Quinoline ATP synthase Inhibitors Drives Antibacterial Activity Against *Pseudomonas aeruginosa***

**Authors:**
Katie T. Ward,[1] Alexander P. L. Williams,[1] Courtney A. Blair,[1] Ananya M. Chatterjee,[1] Abirami Karthikeyan,[1] Addison S. Roper,[1] Casey N. Kellogg,[1] P. Ryan Steed,[1]* Amanda L. Wolfe. [1]*

[1]Department of Chemistry and Biochemistry, University of North Carolina Asheville, One University Heights, Asheville, North Carolina, 28804, United States

**Table of Contents**

Experimental Procedures for compounds **5**-**22** S2

Biological Evaluation of compounds **5**-**22** S8

NMR Spectra for compounds **5**-**22** S11

Table S1. Electron Transport Chain Controls for *E. coli* DK8 pASH20 and PA S29

Table S2. Physical Properties for **1**, **2**, and **5**-**22** S30

**Experimental Procedures:**
**Synthesis and Spectroscopic Data.**
*General*. Reagents and solvents were purchased reagent-grade and used without further purification. All reactions were performed in flame-dried glassware under an Ar or $N_2$ atmosphere. Evaporation and concentration *in vacuo* was performed at 40-45 °C. TLC was conducted using precoated $SiO_2$ 60 F254 glass plates from EMD with visualization by UV light (254 or 366 nm). NMR ($^1H$ or $^{13}C$) were recorded on an Varian INOVA-400 MHz spectrometer or a Bruker Avance-400 MHz spectrometer at 298 K. Residual solvent peaks were used as an internal reference ($CDCl_3$ with 0.1% TMS). Coupling constants (*J*) (H,H) are given in Hz. Coupling patterns are designated as singlet (s), doublet (d), triplet (t), multiplet (m) or quintet. IR spectra were recorded on a Shimadzu IRSpirit FT-IR spectrophotometer and measured neat. Low-resolution mass spectral data were acquired on a Shimadzu single quadrupole LCMS-2020. High-resolution mass spectral Samples were analyzed with a Q Exactive HF-X (ThermoFisher, Bremen, Germany) mass spectrometer. Samples were introduced via a heated electrospray source (HESI) at a flow rate of 10 µL/min. HESI source conditions were set as: nebulizer temperature 400 °C, sheath gas (nitrogen) 20 arb, auxiliary gas (nitrogen) 0 arb, sweep gas (nitrogen) 0 arb, capillary temperature 320 degrees C, RF voltage 45 V. The mass range was set to 100-1000 m/z. All measurements were recorded at a resolution setting of 120,000. Solutions were analyzed at 0.1 mg/mL or less based on responsiveness to the ESI mechanism. Xcalibur (ThermoFisher, Breman, Germany) was used to analyze the data. Molecular formula assignments were determined with Molecular Formula Calculator (v 1.3.0). All observed species were singly charged, as verified by unit *m/z* separation between mass spectral peaks corresponding to the $^{12}C$ and $^{13}C^{12}C_{c-1}$ isotope for each elemental composition.

***HPLC purity.*** Purity analysis of compounds **5**-**22** was conducted on a Shimadzu single quadrupole LCMS-2020 system using a Kinetex 2.6 μm C18 100 Å LC Column 30 x 2.1 mm with a 254 nm detection wavelength. Compounds were prepared in 51 μg/mL solution of LCMS grade acetonitrile (20%) and water (80%). 2 mL of sample were injected at a flow rate of 0.5 mL/min. Gradient elution of 20%-80% acetonitrile with 0.1% formic acid in water with 0.1 % formic acid over 9 minutes.

***Safety Statement.*** No chemical safety hazards were encountered during synthetic experiments of this research work.

***General procedure for reductive amination.*** The methyl sulfide or benzyl sulfide quinoline (1 eq) and amine (1.2 eq) were dissolved in anhydrous methanol (0.09 M) under inert conditions. *N,N*-Diisopropylethylamine (3 eq) was then added dropwise, and the reaction was allowed to stir at 23 °C for 24 h. $NaBH_4$ (2 eq) was then added. After 1 h, the reaction was diluted with DI $H_2O$ and extracted with dichloromethane (2x) or ethyl acetate (3x). The organic layers were then combined, dried over $Na_2SO_4$, and concentrated under reduced pressure. Flash chromatography of the crude extracts ($SiO_2$, 3 × 10 cm, 0−100% $CH_3OH/CH_2Cl_2$ gradient elution) provided the desired products.

*$N^1$,$N^1$-dimethyl-$N^3$-((2-(methylthio)quinolin-3-yl)methyl)propane-1,3-diamine* **5**. General reductive amination procedure starting from **3** (200 mg, 0.984 mmol) and *N*,*N*-Dimethyl-1,3-propanediamine afforded compound **5** (120 mg, 42%) as a sticky translucent solid. $^1$H NMR ($CDCl_3$, 400 MHz): δ 7.93 (d, *J* = 8.4 Hz, 1H), 7.90 (s, 1H), 7.71 (d, *J* = 8.0 Hz, 1H), 7.61-7.57 (m, 1H), 7.40 (t, *J* = 7.2 Hz, 1H), 3.91 (s, 2H), 2.73-2.71 (m, *J* = 3.5 Hz, 5H), 2.36 (t, *J* = 7.2 Hz, 2H), 2.23 (s, 6H), 1.73 (t, *J* = 7.1 Hz, 2H). $^{13}$C NMR ($CDCl_3$, 100 MHz): δ 159.1, 147.3, 133.6, 131.3, 129.1, 127.70, 127.5, 125.9, 125.3, 58.1, 50.2, 48.0, 45.6 (2C), 27.9, 13.0. IR (film) $\nu_{max}$ 3295, 2928, 2857, 2813, 2767, 1614, 1599, 1558, 1489, 1459, 1396, 1328, 1313, 1135, 1044, 751 cm$^{-1}$. HRMS (ESI) *m/z* $[M+H]^+$ calcd for $C_{16}H_{24}N_3S$ 290.1685; found 290.16794 .

*$N^1$-((2-(benzylthio)quinolin-3-yl)methyl)-$N^3$,$N^3$-dimethylpropane-1,3-diamine* **6**. General reductive amination procedure starting from **4** (100 mg, 0.358 mmol) and *N*,*N*-Dimethyl-1,3-propanediamine afforded compound **6** (60 mg, 46%) as a translucent solid. $^1$H NMR ($CDCl_3$, 400 MHz): δ 7.98 (d, *J* = 8.4 Hz, 1H), 7.94 (s, 1H), 7.72 (d, *J* = 8.0 Hz, 1H), 7.63 (t, *J* = 7.6 Hz, 1H), 7.49 (d, *J* = 7.3 Hz, 2H), 7.42 (t, *J* = 7.6 Hz, 1H), 7.29 (t, *J* = 7.4 Hz, 2H), 7.23 (d, *J* = 7.2 Hz, 1H), 4.66 (s, 2H), 3.89 (s, 2H), 2.70 (t, *J* = 6.9 Hz, 2H), 2.34 (t, *J* = 7.1 Hz, 2H), 2.22 (s, 6H) 1.71 (d, *J* = 14.2 Hz, 2H). $^{13}$C NMR ($CDCl_3$, 100 MHz): δ 158.3, 147.2, 138.5, 134.0, 131.3, 129.5 (2C), 129.3, 128.6 (2C), 127.8, 127.6, 127.2, 126.3, 125.5, 58.1, 50.3, 48.1, 45.6 (2C), 34.2, 28.0. IR (film) $\nu_{max}$ 2929, 2768, 1616, 1596, 1555, 1489, 1452, 1395, 1324, 1133, 1117, 1070, 1041, 749, 696, 600 cm$^{-1}$. HRMS (ESI) *m/z* $[M+H]^+$ calcd for $C_{22}H_{28}N_3S$ 366.1998; found 366.19916 .

*$N^1$,$N^1$-dimethyl-$N^4$-((2-(methylthio)quinolin-3-yl)methyl)butane-1,4-diamine* **7**. General reductive amination procedure starting from **3** (200 mg, 0.984 mmol) and *N*,*N*-Dimethyl-1,4-butanediamine afforded compound **7** (130 mg, 44%) as an off white solid. $^1$H NMR ($CDCl_3$, 400 MHz): δ 7.93 (d, *J* = 8.4 Hz, 1H), 7.88 (s, 1H), 7.70 (d, *J* = 8.0 Hz, 1H), 7.60 (m, *J* = 4.1 Hz, 1H), 7.39 (m, *J* = 3.9 Hz, 1H), 3.90 (s, 2H), 2.70-2.66 (m, 5H), 2.25 (t, *J*= 7.1 Hz, 2H), 2.20 (s, 6H), 1.57-1.50 (m, 4H). $^{13}$C NMR ($CDCl_3$, 100 MHz): δ 159.1, 147.3, 133.5, 131.5, 129.1, 127.7, 127.5, 125.9, 125.3, 59.8, 50.2, 49.5, 45.6 (2C), 28.1, 25.7, 13.0. IR (film) $\nu_{max}$ 2929, 2859, 2813, 2777, 2767, 1616, 1599, 1558, 1489, 1458, 1403, 1327, 1313, 1135, 1043, 751 cm$^{-1}$. HRMS (ESI) *m/z* $[M+H]^+$ calcd for $C_{17}H_{26}N_3S$ 304.1842; found 304.18359 .

*$N^1$-((2-(benzylthio)3uinoline-3-yl)methyl)-$N^4$,$N^4$-dimethylbutane-1,4-diamine* **8**. General reductive amination procedure starting from **4** (100 mg, 0.358 mmol) and *N*,*N*-Dimethyl-1,4-butanediamine afforded compound **8** (104 mg, 77%) as clear semi-solid. $^1$H NMR (400 MHz, $CDCl_3$) δ 7.98 (d, *J* = 8.4 Hz, 1H), 7.92 (s, 1H), 7.72 (d, *J* = 7.9 Hz, 1H), 7.62 (t, *J* = 8.3 Hz, 1H), 7.49 (d, *J* = 7.2 Hz, 2H), 7.42 (t, *J* = 7.5 Hz, 1H), 7.29 (t, *J* = 7.4 Hz, 2H), 7.22 (q, *J* = 6.5 Hz, 1H), 4.66 (s, 2H), 3.88 (s, 2H), 2.66 (t, *J* = 6.6 Hz, 2H), 2.25 (t, *J* = 6.8 Hz, 2H), 2.19 (s, 6H), 1.52 (m, 4H). $^{13}$C NMR ($CDCl_3$, 100 MHz): δ 158.3, 147.1, 138.5, 133.8, 131.5, 129.4 (2C), 129.2, 128.6 (2C), 127.8, 127.57, 127.2, 126.2, 125.4, 59.8, 50.2, 49.5, 45.6 (2C), 34.1, 28.1, 25.6. IR (film) $\nu_{max}$ 2935, 2859, 2814, 2777, 1617, 1597, 1558, 1489, 1453, 1396, 1326, 1135, 1041, 751 cm$^{-1}$. HRMS (ESI) *m/z* $[M+H]^+$ calcd for $C_{23}H_{30}N_3S$ 380.2155; found 380.21539 .

*$N^1$,$N^1$-dimethyl-$N^5$-((2-(methylthio)quinolin-3-yl)methyl)pentane-1,5-diamine* **9**. General reductive amination procedure starting from **3** (200 mg, 0.984 mmol) and *N*,*N*-dimethylpentane-

1,5-diamine afforded compound **9** (37 mg, 12%) as an sticky off-white solid. $^{1}H$ NMR ($CDCl_3$, 400 MHz): δ $^{1}H$ NMR (400 MHz, cdcl$_3$) δ 7.96 (s, 1H), 7.93 (d, *J* = 8.4 Hz, 1H), 7.74 (d, *J* = 7.9 Hz, 1H), 7.62-7.59 (m, 1H), 7.41 (t, *J* = 7.1 Hz, 1H), 3.94 (s, 2H), 2.71-2.67 (m, 5H), 2.40 (t, *J* = 7.6 Hz, 2H), 2.32 (s, 6H), 1.57 (m, *J* = 6.9 Hz, 4H), 1.40-1.33 (m, *J* = 7.3 Hz, 2H). $^{13}C$ NMR ($CDCl_3$, 100 MHz): δ 159.2, 147.4, 133.9, 131.0, 129.3, 127.8, 127.7, 126.0, 125.4, 59.3, 50.1, 49.3, 45.0 (2C), 29.7, 26.9, 25.0, 13.1. IR (film) $\nu_{max}$ 3394, 2928, 2860, 2813, 2768, 1599, 1461, 1396, 1313, 1135, 1044, 752 cm$^{-1}$. HRMS (ESI) *m/z* $[M+H]^+$ calcd for $C_{18}H_{28}N_3S$ 318.1998; found 318.19955.

*$N^1$-((2-(benzylthio)quinolin-3-yl)methyl)-$N^5$,$N^5$-dimethylpentane-1,5-diamine* **10**. General reductive amination procedure starting from **4** (100 mg, 0.358 mmol) and *N*,*N*-dimethylpentane-1,5-diamine afforded compound **10** (32 mg, 23%) as yellow semi-solid. $^{1}H$ NMR (400 MHz, $CDCl_3$) δ 7.98 (d, *J* = 8.4 Hz, 1H), 7.95 (s, 1H), 7.74 (d, *J* = 8.0 Hz, 1H), 7.63 (t, *J* = 7.6 Hz, 1H), 7.49 (d, *J* = 7.3 Hz, 2H), 7.42 (t, *J* = 7.5 Hz, 1H), 7.29 (t, *J* = 7.4 Hz, 2H), 7.23 (q, *J* = 7.1 Hz, 1H), 4.66 (s, 2H), 3.90 (s, 2H), 2.65 (t, *J* = 7.1 Hz, 2H), 2.30 (t, *J* = 7.5 Hz, 2H), 2.24 (s, 6H) 1.52 (m, *J* = 7.7 Hz, 4H), 1.35 (q, *J* = 7.7 Hz, 2H) . $^{13}C$ NMR ($CDCl_3$, 100 MHz): δ 158.3, 147.2, 138.5, 134.0, 131.3, 129.5 (2C), 129.3, 128.6 (2C), 127.8, 127.7, 127.2, 126.3, 125.5, 59.7, 50.2, 49.5, 45.4 (2C), 34.2, 30.0, 27.4, 25.2. IR (film) $\nu_{max}$ 3400, 2932, 2856, 2815, 2771, 1617, 1597, 1558, 1489, 1453, 1396, 1372, 1135, 1041, 752, 698 cm$^{-1}$. HRMS (ESI) *m/z* $[M+H]^+$ calcd for $C_{24}H_{32}N_3S$ 394.2311; found 394.23056.

*N,N-dimethyl-4-((((2-(methylthio)quinolin-3-yl)methyl)amino)methyl)aniline* **11**. General reductive amination procedure starting from **3** (100 mg, 0.492 mmol) and 4-dimethylaminobenzylamine dihydrochloride afforded compound **11** (80 mg, 48%) as a dark yellow sticky solid. $^{1}H$ NMR (400 MHz, $CDCl_3$) δ 7.94 (d, *J* = 8.4 Hz, 1H), 7.91 (s, 1H), 7.70 (d, *J* = 8.0 Hz, 1H), 7.60 (t, *J* = 7.6 Hz, 1H), 7.40 (t, *J* = 7.5 Hz, 1H), 7.24 (d, *J* = 8.4 Hz, 2H), 6.72 (d, *J* = 8.5 Hz, 2H), 3.92 (s, 2H), 3.76 (s, 2H), 2.92 (s, 6H), 2.71 (s, 3H) . $^{13}C$ NMR ($CDCl_3$, 100 MHz): δ 159.3, 150.0, 147.3, 133.7, 131.4, 129.4 (2C), 129.3, 127.9, 127.7, 127.6, 126.0, 125.3, 112.9 (2C), 52.9, 49.4, 40.9 (2C), 13.1. IR (film) $\nu_{max}$ 2923, 2851, 2791, 1614, 1522, 1488, 1443, 1395, 1383, 1346, 1331, 1313, 1346, 1331, 1205, 1165, 1135, 1045, 948, 781, 752 cm$^{-1}$. HRMS (ESI) *m/z* $[M+H]^+$: calcd for $C_{20}H_{24}N_3S$ 338.1685; found 338.16782.

*4-((((2-(benzylthio)quinolin-3-yl)methyl)amino)methyl)-N,N-dimethylaniline* **12**. General reductive amination procedure starting from **4** (50 mg, 0.179 mmol) and 4-dimethylaminobenzylamine dihydrochloride afforded compound **12** (26 mg, 35%) as orange/brown semi solid. $^{1}H$ NMR ($CDCl_3$, 400 MHz): δ 7.98 (d, *J* = 8.4 Hz, 1H), 7.95 (s, 1H), 7.72 (d, J = 8.0 Hz, 1H), 7.63 (m, 1H), 7.49 (d, J = 7.2 Hz, 2H), 7.42 (t, J = 7.1 Hz, 1H), 7.29 (t, J = 7.4 Hz, 2H), 7.24-7.20 (m, 3H), 6.71 (d, J = 8.6 Hz, 2H), 4.66 (s, 2H), 3.91 (s, 2H), 3.74 (s, 2H), 2.92 (s, 6H). $^{13}C$ NMR ($CDCl_3$, 100 MHz): δ. 158.4, 150.1, 147.2, 138.6, 134.0, 131.4, 129.5 (2C), 129.4 (2C), 129.3, 128.6 (2C), 128.0, 127.8, 127.6, 127.2, 126.3, 125.5, 112.9 (2C), 53.0, 49.4, 41.0 (2C), 34.2 cm$^{-1}$. IR (film) $\nu_{max}$ 2925, 1614, 1597, 1522, 1491, 1452, 1349, 1330, 1135, 1043, 752 cm$^{-1}$. HRMS (ESI) *m/z* $[M+H]^+$: calcd for $C_{26}H_{28}N_3S$ 414.1998; found 414.19925.

*2-(1-ethylpiperidin-4-yl)-N-((2-(methylthio)quinolin-3-yl)methyl)ethan-1-amine* **13**. General reductive amination procedure starting from **3** (200 mg, 0.984 mmol) and 2-(1-ethylpiperidin-4-

yl)ethanamine afforded compound **13** (177 mg, 52%) as an off white solid. $^1$H NMR ($CDCl_3$, 400 MHz): δ 7.93 (d, *J* = 8.3 Hz, 1H), 7.85 (s, 1H), 7.69 (d, *J* = 7.9 Hz, 1H), 7.59 (t, *J* = 7.5 Hz, 1H), 7.39 (t, *J* = 7.3 Hz, 1H), 3.87 (s, 2H), 2.94 (d, *J* = 10.6 Hz, 2H), 2.70 (s, 3H), 2.66 (t, *J* = 7.2 Hz, 2H), 2.40 (q, *J* = 6.9 Hz, 2H), 1.88 (t, *J* = 10.3 Hz, 2H), 1.67 (d, *J* = 10.1 Hz, 2H), 1.48 (d, *J* = 5.9 Hz, 2H), 1.33-1.29 (m, *J* = 9.3 Hz, 4H), 1.08 (t, *J* = 7.0 Hz, 3H). $^{13}$C NMR ($CDCl_3$, 100 MHz): δ 158.9, 147.1, 133.3, 131.2, 129.0, 127.6, 127.3, 125.7, 125.1, 53.3 (2C), 52.5, 50.2, 46.8, 36.7, 33.6, 32.0 (2C), 12.8, 11.9. IR (film) $\nu_{max}$ cm$^{-1}$ 2917, 2801, 1597, 1558, 1452, 1380, 1311, 1135, 1047, 751. HRMS (ESI) *m/z* [M+H]$^+$ calcd for $C_{20}H_{30}N_3S$ 344.2155; found 344.21494.

*N-((2-(benzylthio)quinolin-3-yl)methyl)-2-(1-ethylpiperidin-4-yl)ethan-1-amine* **14**. General reductive amination procedure starting from **4** (100 mg, 0.358 mmol) and 2-(1-ethylpiperidin-4-yl)ethanamine afforded compound **14** (43 mg, 28%) as brown solid. $^1$H NMR (400 MHz, $CDCl_3$) δ 7.99 (d, *J* = 8.4 Hz, 1H), 7.91 (s, 1H), 7.72 (d, *J* = 8.0 Hz, 1H), 7.63 (t, *J* = 7.6 Hz, 1H), 7.49 (d, *J* = 7.3 Hz, 2H), 7.43 (t, *J* = 7.4 Hz, 1H), 7.25 (d, *J* = 19.8 Hz, 2H), 7.25 (t, *J* = 5.2 Hz, 1H), 4.66 (s, 2H), 3.88 (s, 2H), 2.91 (d, *J* = 11.5 Hz, 2H), 2.66 (t, *J* = 7.3 Hz, 2H), 2.37 (q, *J* = 7.2 Hz, 2H), 1.84 (t, *J* = 11.3 Hz, 2H), 1.66 (d, *J* = 12.0 Hz, 2H), 1.47 (m, 2H), 1.31 (m, 4H), 1.07 (t, *J* = 7.2 Hz, 3H). $^{13}$C NMR ($CDCl_3$, 100 MHz): δ 158.3, 147.2, 138.5, 134.0, 131.5, 129.5 (2C), 129.3, 128.6 (2C), 127.8, 127.6, 127.2, 126.2, 125.5, 53.6 (2C), 52.8, 50.5, 47.0, 37.0, 34.2, 33.9, 32.5 (2C), 12.8. IR (film) $\nu_{max}$ cm$^{-1}$ 2925, 2803, 2765, 1617, 1597, 1558, 1489, 1472, 1456, 1395, 1327, 1135, 1045, 752, 698. HRMS (ESI) *m/z* [M+H]$^+$ calcd for $C_{26}H_{34}N_3S$ 420.2468; found 420.24643.

*2-(1-cyclopentylpiperidin-4-yl)-N-((2-(methylthio)quinolin-3-yl)methyl)ethan-1-amine* **15**. General reductive amination procedure starting from **3** (100 mg, 0.492 mmol) and 2-(1-cyclopentylpiperidin-4-yl)ethanamine afforded compound **15** (121.3 mg, 64%) as a very sticky translucent solid. $^1$H NMR (400 MHz, $CDCl_3$) δ 7.92 (d, *J* = 8.4 Hz, 1H), 7.86 (s, 1H), 7.69 (d, *J* = 7.9 Hz, 1H), 7.59 (m, *J* = 4.1 Hz, 1H), 7.39 (t, *J* = 7.5 Hz, 1H), 3.88 (s, 2H), 3.02 (d, *J* = 11.5 Hz, 2H), 2.70 (s, 3H), 2.66 (t, *J* = 7.3 Hz, 2H), 2.46 (t, *J* = 8.0 Hz, 1H), 1.92 (t, *J* = 11.0 Hz, 2H), 1.83 (m, 2H), 1.65 (m, 4H), 1.50-1.40 (m, 10H). $^{13}$C NMR ($CDCl_3$, 100 MHz): δ 159.1, 147.3, 133.6, 131.4, 129.1, 127.7, 127.5, 125.9, 125.3, 67.8, 52.8 (2C), 50.4, 46.9, 36.8, 33.7, 32.3 (2C), 30.4 (2C), 24.2 (2C), 13.0. IR (film) $\nu_{max}$ 2952, 2867, 2861, 2847, 2823, 2798, 2749, 1597, 1558, 1446, 1395, 1383, 1354, 1327, 1311, 1135, 1119, 1045, 956, 749, 731, 599 cm$^{-1}$. HRMS (ESI) *m/z* [M+H]$^+$: calcd for $C_{23}H_{34}N_3S$ 384.2468; found 384.24564.

*N-((2-(benzylthio)quinolin-3-yl)methyl)-2-(1-cyclopentylpiperidin-4-yl)ethan-1-amine* **16**. General reductive amination procedure starting from **4** (100 mg, 0.358 mmol) and 2-(1-cyclopentylpiperidin-4-yl)ethanamine afforded compound **16** (26 mg, 16%) as yellow solid. $^1$H NMR (400 MHz, $CDCl_3$) δ 7.98 (d, *J* = 8.4 Hz, 1H), 7.96 (s, 1H) 7.75 (d, *J* = 8.0 Hz, 1H), 7.66 (m,1H), 7.49 (d, *J* = 7.2 Hz, 2H), 7.45 (t, *J* = 7.0 Hz, 1H), 7.31 (t, *J* = 7.6 Hz, 2H), 7.24 (m, 1H), 4.66 (s, 2H), 3.92 (s, 2H), 3.47 (s, 2H), 3.05 (s, 1H), 2.70 (t, *J* = 6.8 Hz, 2H), 2.43 (s, 2H), 2.02 (m, 5H), 1.88 (t, *J* = 6.9 Hz, 6H), 1.57 (m, 5H). $^{13}$C NMR ($CDCl_3$, 100 MHz, 273K) δ 158.4, 147.3, 138.6, 134.2, 131.3, 129.5 (2C), 129.4, 128.6 (2C), 127.8, 127.6, 127.3, 126.2, 125.6, 68.0, 52.5 (2C), 50.6, 46.6, 36.1, 34.3, 32.7 (2C), 30.6 (2C), 29.4, 24.1 (2C). IR (film) $\nu_{max}$ 3390, 2923, 2656, 1617, 1596, 1489, 1452, 1397, 1328, 1135, 1047, 920, 754, 728 cm$^{-1}$. HRMS (ESI) *m/z* [M+H]$^+$: calcd for $C_{29}H_{38}N_3S$ 460.2781; found 460.27725.

*2-(1-methyl-1H-pyrrol-2-yl)-N-((2-(methylthio)quinolin-3-yl)methyl)ethan-1-amine* **17**. General reductive amination procedure starting from **3** (200 mg, 0.984 mmol) and 2-(1-methyl-1H-pyrrol-2-yl)ethanamine afforded compound **17** (86 mg, 28%) as an off white solid solid. $^1$H NMR (400 MHz, $CDCl_3$) δ 8.00 (d, *J* = 8.3 Hz, 1H), 7.88 (s, 1H) 7.75 (d, *J* = 8.0 Hz, 1H), 7.66 (t, *J* = 7.5 Hz, 1H), 7.45 (t, *J* = 7.4 Hz, 1H), 6.61 (s, 1H), 6.13 (s, 1H) 6.00 (s, 1H), 3.99 (s, 2H), 3.56 (s, 3H). 2.99 (t, *J* = 6.9 Hz, 2H), 2.87 (t, *J* = 6.9 Hz, 2H), 2.77 (s, 3H). $^{13}$C NMR ($CDCl_3$, 100 MHz): δ 159.1, 147.2, 133.3, 131.3, 130.7, 129.1, 127.7, 127.5, 125.9, 125.3, 121.6, 106.8, 106.3, 49.9, 48.4, 33.7, 26.9, 13.0. IR (film) $\nu_{max}$ 2922, 2823, 1614, 1597, 1555, 1489, 1452, 1395, 1326, 1311, 1297, 1168, 1135, 1117, 1087, 1044, 955, 909, 859, 811, 778, 749, 699, 599 cm$^{-1}$. HRMS (ESI) *m/z* $[M+H]^+$: calcd for $C_{18}H_{22}N_3S$ 312.1529; found 312.15237.

*N-((2-(benzylthio)quinolin-3-yl)methyl)-2-(1-methyl-1H-pyrrol-2-yl)ethan-1-amine* **18**. General reductive amination procedure starting from **4** (200 mg, 0.716 mmol) and 2-(1-methyl-1H-pyrrol-2-yl)ethanamine afforded compound **18** (64 mg, 23%) as brown semi-solid. $^1$H NMR (400 MHz, $CDCl_3$) δ 7.98 (d, *J* = 8.1 Hz, 1H), 7.86 (s, 1H), 7.71 (d, *J* = 7.7 Hz, 1H), 7.63 (t, *J* = 7.1 Hz, 1H), 7.49 (d, *J* = 6.9 Hz, 2H), 7.42 (t, *J* = 7.1 Hz, 1H), 7.30 (t, *J* = 6.8 Hz, 2H), 7.24 (d, *J* = 6.3 Hz, 1H), 6.54 (s, 1H), 6.05 (s, 1H), 5.92 (s, 1H), 4.66 (s, 2H), 3.91 (s, 2H), 3.50 (s, 3H) 2.91 (m, 2H), 2.80 (m, 2H). $^{13}$C NMR ($CDCl_3$, 100 MHz): δ158.3, 147.2, 138.5, 133.8, 131.2, 130.7, 129.5 (2C), 129.3, 128.6 (2C), 127.8, 127.6, 127.3, 126.2, 125.5, 121.7, 106.9, 106.4, 50.0, 48.4, 34.2, 33.8, 27.0. IR (film) $\nu_{max}$ 2922, 2836, 1616, 1594, 1558, 1491, 1452, 1396, 1324, 1297, 1203, 1169, 1133, 1117, 1041, 909, 751, 699, 600 cm$^{-1}$. HRMS (ESI) *m/z* $[M+H]^+$: calcd for $C_{24}H_{26}N_3S$ 388.1842; found 388.18372.

*2-(4,6-dimethylpyrimidin-2-yl)-N-((2-(methylthio)quinolin-3-yl)methyl)ethan-1-amine* **19**. General reductive amination procedure starting from **3** (200 mg, 0.984 mmol) afforded compound **19** (85 mg, 22%) as an off white solid. $^1$H NMR (400 MHz, $CDCl_3$) δ 7.96 (s, 1H), 7.93 (d, *J* = 8.4 Hz, 1H), 7.71 (d, *J* = 8.0 Hz, 1H), 7.61 (t, *J* = 7.2 Hz, 1H), 7.40 (t, *J* = 7.2 Hz, 1H), 6.85 (s, 1H), 4.01 (s, 2H), 3.18 (m, 5H), 2.70 (s, 3H), 2.42 (s, 6H). $^{13}$C NMR ($CDCl_3$, 100 MHz): δ 168.8, 166.7 (2C), 159.1, 147.4, 133.9, 130.8, 129.2, 127.7, 127.6, 126.0, 125.3, 117.8, 49.8, 47.7, 38.9, 24.1 (2C), 13.0. IR (film) $\nu_{max}$ 2923, 2851, 1590, 1553, 1489, 1436, 1382, 1311, 1261, 1170, 1135, 1043, 910, 751, 729, 541 cm$^{-1}$. HRMS (ESI) *m/z* $[M+H]^+$: calcd for $C_{19}H_{23}N_4S$ 339.1638; found 339.16367.

*N-((2-(benzylthio)quinolin-3-yl)methyl)-2-(4,6-dimethylpyrimidin-2-yl)ethan-1-amine* **20**. General reductive amination procedure starting from **4** (200 mg, 0.716 mmol) and 2-(4,6-dimethylpyrimidin-2-yl)ethanamine dihydrochloride afforded compound **20** (138 mg, 46%) as brown semi-solid. $^1$H NMR (400 MHz, $CDCl_3$) δ 7.97 (d, *J* = 8.4 Hz, 1H), 7.94 (s, 1H), 7.68 (d, *J* = 8.0 Hz, 1H), 7.60 (t, *J* = 7.6 Hz, 1H), 7.47 (d, *J* = 7.5 Hz, 2H), 7.39 (t, *J* = 7.5 Hz, 1H), 7.28 (t, *J* = 7.4 Hz, 2H), 7.22 (t, *J* = 7.1 Hz, 1H), 6.76 (s, 1H), 4.63 (s, 2H), 3.93 (s, 2H), 3.14 (m, 4H), 2.37 (s, 6H). $^{13}$C NMR ($CDCl_3$, 100 MHz): δ 168.8, 166.6 (2C), 158.2, 147.1, 138.3, 133.8, 131.1, 129.4 (2C), 129.1, 128.5 (2C), 127.6, 127.5, 127.1, 126.1, 125.3, 117.6, 49.8, 47.7, 39.1, 34.0, 24.0 (2C). IR (film) $\nu_{max}$ 3027, 2956, 2843, 1589, 1551, 1489, 1442, 1395, 1324, 1133, 1117, 1041, 955, 806, 780, 751, 698, 600, 541 cm$^{-1}$. HRMS (ESI) *m/z* $[M+H]^+$: calcd for $C_{25}H_{27}N_4S$ 415.1951; found 415.19492.

*2-(1H-imidazol-5-yl)-N-((2-(methylthio)quinolin-3-yl)methyl)ethan-1-amine* **21**. General reductive amination procedure starting from **3** (200 mg, 0.984 mmol) and histamine and and 2-(4,6-dimethylpyrimidin-2-yl)ethanamine dihydrochloride afforded compound **21** (167.5 mg, 57%) as a yellow solid. $^{1}$H NMR (400 MHz, $CDCl_3$) δ 7.91 (d, *J* = 8.4 Hz, 1H), 7.82 (s, 1H), 7.66 (d, *J* = 7.9 Hz, 1H), 7.58 (t, *J* = 7.5 Hz, 1H), 7.52 (s, 1H), 7.37 (t, *J* = 7.4 Hz, 1H), 6.80 (br, 1NH), 6.75 (s, 1H), 3.91 (s, 2H), 2.94 (t, *J* = 6.0 Hz, 2H), 2.80 (t, *J* = 5.7 Hz, 2H), 2.68 (s, 3H). $^{13}$C NMR ($CDCl_3$, 100 MHz): δ 159.0, 147.3, 134.6, 134.2, 134.0, 130.4, 129.4, 127.7, 127.5, 125.8, 125. 5, 118.7, 50.1, 48.7, 26.3, 13.0. IR (film) $\nu_{max}$ 3078, 2925, 2840, 1614, 1597, 1558, 1489, 1456, 1397, 1330, 1313, 1261, 1136, 1104, 1045, 910, 813, 780, 752, 731, 624 $cm^{-1}$. HRMS (ESI) *m/z* $[M+H]^+$: calcd for $C_{16}H_{19}N_4S$ 299.1325; found 299.13229.

*N-((2-(benzylthio)quinolin-3-yl)methyl)-2-(1H-imidazol-5-yl)ethan-1-amine* **22**. General reductive amination procedure starting from **4** (50 mg, 0.178 mmol) and histamine afforded compound **22** (31 mg, 46%) as yellow semi-solid. δ$^{1}$H NMR (400 MHz, $CDCl_3$) δ 8.00 (d, *J* = 8.4 Hz, 1H), 7.86 (s, 1H), 7.71 (d, *J* = 8.1 Hz, 1H), 7.65 (m, 1H), 7.50 (d, *J* = 7.1 Hz, 2H), 7.44 (t, *J* = 7.9 Hz, 1H), 7.28 (m, 4H), 6.74 (s, 1H), 4.68 (s, 2H), 3.93 (br, 1H), 2.93 (t, *J* = 6.0 Hz, 2H), 2.77 (t, *J* = 5.9 Hz, 2H). $^{13}$C NMR ($CDCl_3$, 100 MHz): δ. 158.39, 147.36, 138.5, 134.8, 134.5, 133.3, 130.7, 129.6 (2C), 129.5, 128.8 (2C), 127.9, 127.6, 127.4, 126.1, 125.7, 120.5, 50.9, 48.9, 34.1, 26.0. IR (film) $\nu_{max}$ 3083, 3055, 2953, 2919, 2846, 1616, 1596, 1558, 1489, 1452, 1397, 1328, 1135, 1106, 1044, 752, 699 $cm^{-1}$. HRMS (ESI) *m/z* $[M+H]^+$: calcd for $C_{22}H_{23}N_4S$ 375.1638; found 375.16342.

**Biological Evaluation of Lead compounds.**
Compounds **5**-**22** were > 95% via HPLC (see HPLC purity for method).

**Representative LCMS trace of Compound 14 (M + H = 420 m/z):**

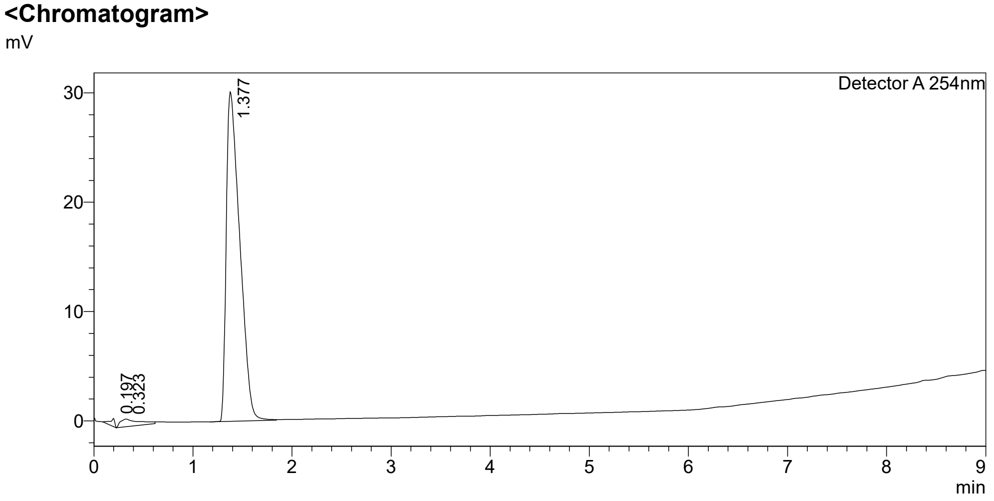


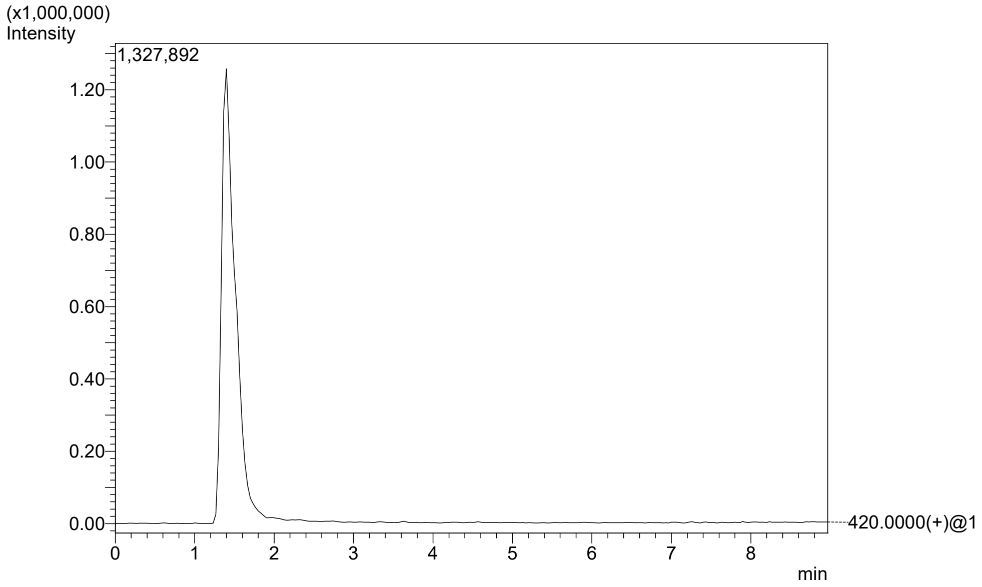


***Safety statement.***
**Caution!** Experiments utilizing Pseudomonas aeruginosa strains were handled following BSL 2 protocols.
**Caution!** CCCP and nigericin are ionophores that are toxic if swallowed.

*General Sterilization Procedure.* The following are general steps, unless otherwise noted. All steps were completed with aseptic techniques. All media and glassware were sterilized via autoclave at 121 °C for 60 minutes. All agitation occurred at 160 rpm in a temperature-controlled console shaker (Excella E25) at 37 °C. Full strength tryptic soy broth (TSB) was made by dissolving 30 g BD Bacto TSB powder in 1 L deionized water. Purchased and acquired bacterial strains used were

*Pseudomonas aeruginosa* (ATCC 9027), Drug-Resistant *Pseudomonas aeruginosa* from ATCC Panel MP-23 (BAA-2108, BAA-2109, BAA-2110), and *Pseudomonas aeruginosa* efflux knockout GKCW120 (PΔ6)[14] provided by Zgurskaya and co-workers at the University of Oklahoma.

*Antimicrobial Susceptibility Assay Procedure*. Susceptibility testing was performed in biological triplicate, using the broth microdilution method as outlined by the Clinical and Laboratory Standards Institute. Briefly, minimum inhibitory concentrations (MIC) determinations were carried out in 96-well microtiter plates with 2-fold serial dilutions of the compounds from 0 µg/mL to 128 µg/mL (final assay concentrations) in DMSO. Briefly, to each well 1 µL of compound in DMSO, 89 µL of TSB, and 10 µL of bacterial inoculum, grown from frozen stock in 10 mL of TSB for 4-6 hours, were added. After incubation for 12–15 h at 37 °C, absorbance at 590 nm was read on a Biotek Synergy HTX Multi-mode plate reader. Data was processed by background subtracting the media absorbance and then normalizing the data to full bacterial growth with only vehicle. MIC is defined as the lowest concentration of antibiotic or antibiotic/adjuvant combination that achieves ≥ 85% growth inhibition, which corresponds to no visible growth.

*Preparation of Inverted Membrane Vesicles.* Vesicles were prepared as previously described[10] from PA 9027 cells or *E. coli* DK8 cells (lacking a chromosomal copy of the *unc* operon, which encodes ATP synthase) transformed with the pASH20, a whole operon plasmid that expresses the PA ATP synthase.[11] Briefly, cells were grown in LB liquid medium at 37 °C with shaking and harvested by centrifugation after 7 hours of growth. Transformant *E. coli* cells were grown in the presence of 100 µg/mL ampicillin. Cells were disrupted in TMG buffer (50 mM Tris-HCl, $MgCl_2$, 10% (v/v) glycerol, pH 7.5) using an Avestin B15 homogenizer at 19,000 psi. After unbroken cells and debris were cleared by centrifugation at 9,000 x g, inverted vesicles were collected by centrifugation at 193,000 x g, resuspended in TMG buffer, and stored at -80 °C. Protein concentrations were determined using a modified Lowry assay.

*Determination of ATP synthesis activity.* Inhibition of ATP synthesis activity of *E. coli* DK8/pASH20 inverted vesicles by test compounds was determined using an endpoint assay essentially as previously described.[11] In this assay, the synthesis reaction was initiated by 2.5 mM NADH, proceeded for 10 min, and was stopped with 1% trichloroacetic acid. Samples from each reaction were diluted 500-fold prior to addition of luciferase, since quinoline compounds are known to inhibit luciferase.[11] Each replicate set included a positive control containing DMSO with no compound and a negative control containing carbonyl cyanide m-chlorophenyl hydrazone. Luminescence values were corrected for background by subtracting the negative control and then normalized to the positive control within the same replicate. Corrected, normalized activities at increasing concentrations of test compounds were fit with a dose-response curve (Eq. 1), where *c* is the concentration of compound and *n* is the Hill coefficient, to find the $IC_{50}$. Fits and 95% confidence bands were determined using GraphPad Prism software.

Equation 1:

$$Relative\ Activity = \frac{1}{1 + \left(\frac{c}{IC_{50}}\right)^{n}}$$

*Determination of electron transport activity.* As previously described,[11] inverted membrane vesicles from PA 9027 or *E. coli* DK8 pASH20 were added to HMK buffer containing 0.3 µg/mL 9-amino-6-chloro-2-methoxyacridine (ACMA) and 0-64 µg/mL test compound in DMSO. After measurement of baseline ACMA fluorescence for 60 s, redox-driven $H^+$ pumping was initiated by addition of NADH to 0.8 mM, and fluorescence quenching was monitored for 120 s before addition of nigericin to 0.5 µg/mL. Relative activity is reported as the minimum fluorescence value normalized to the maximum fluorescence value following addition of nigericin.

## NMR Spectra

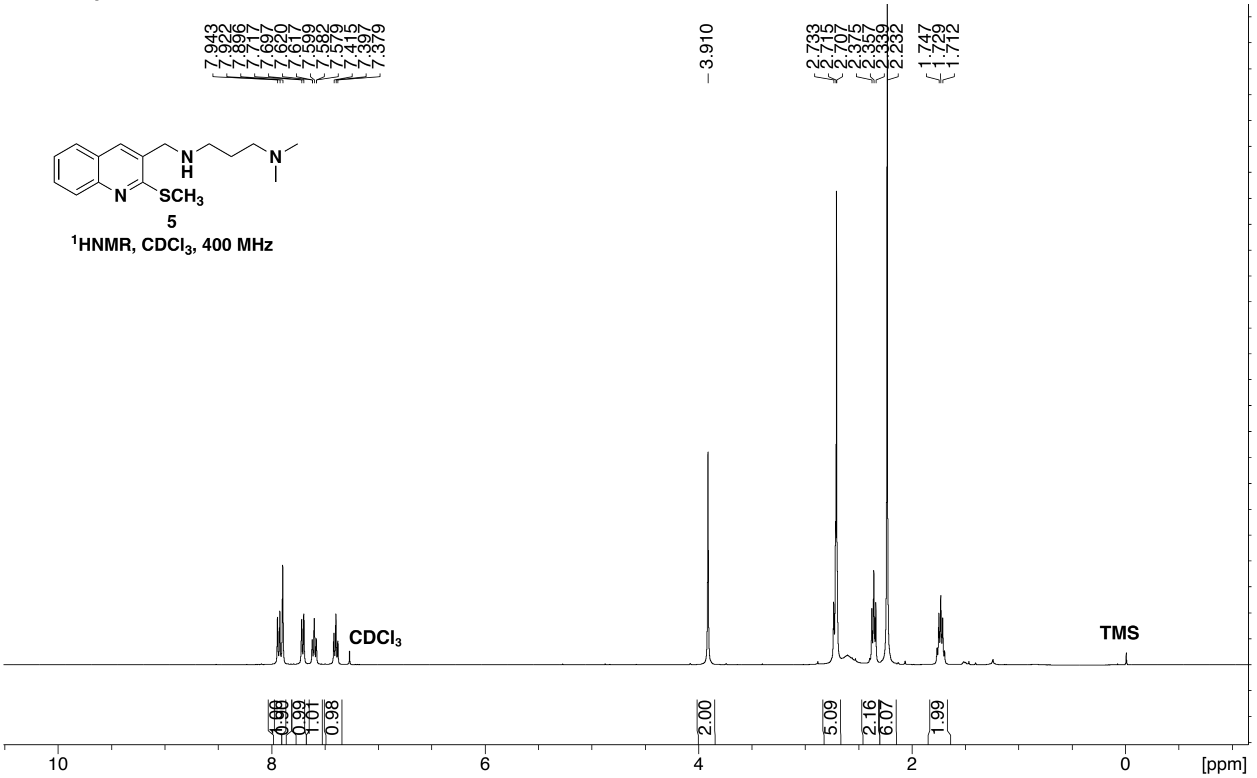
7.943 7.922 7.896 7.717 7.697 7.620 7.617 7.599 7.582 7.579 7.415 7.397 7.379
3.910
2.733 2.715 2.707 2.375 2.357 2.339 2.232
1.747 1.729 1.712
5
$^1$HNMR, $CDCl_3$, 400 MHz
$CDCl_3$
TMS
0.98 1.01 0.99 2.00 5.09 2.16 6.07 1.99
10
8
6
4
2
0
[ppm]

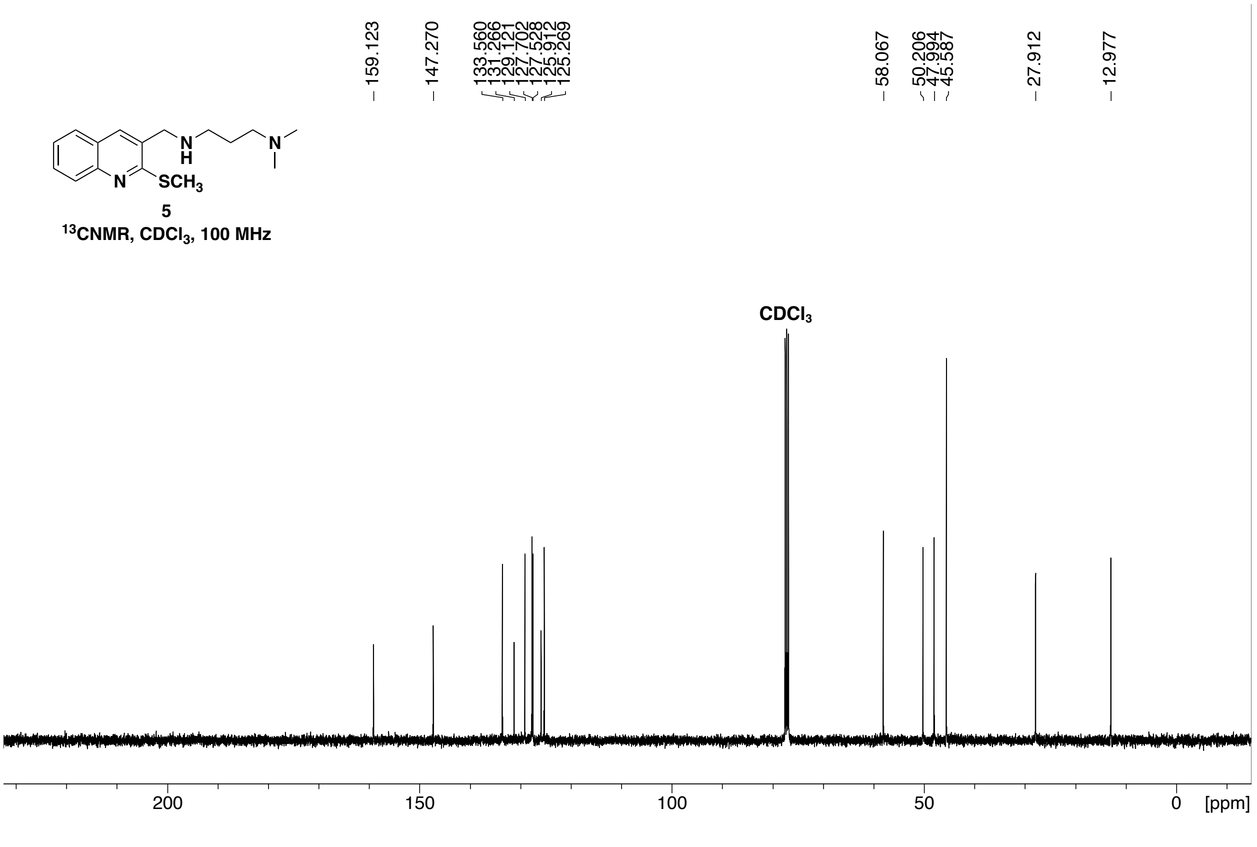
159.123
147.270
133.560 131.266 129.121 127.702 127.528 125.912 125.269
58.067
50.206 47.994 45.587
27.912
12.977
5
$^{13}$CNMR, $CDCl_3$, 100 MHz
$CDCl_3$
200
150
100
50
0
[ppm]

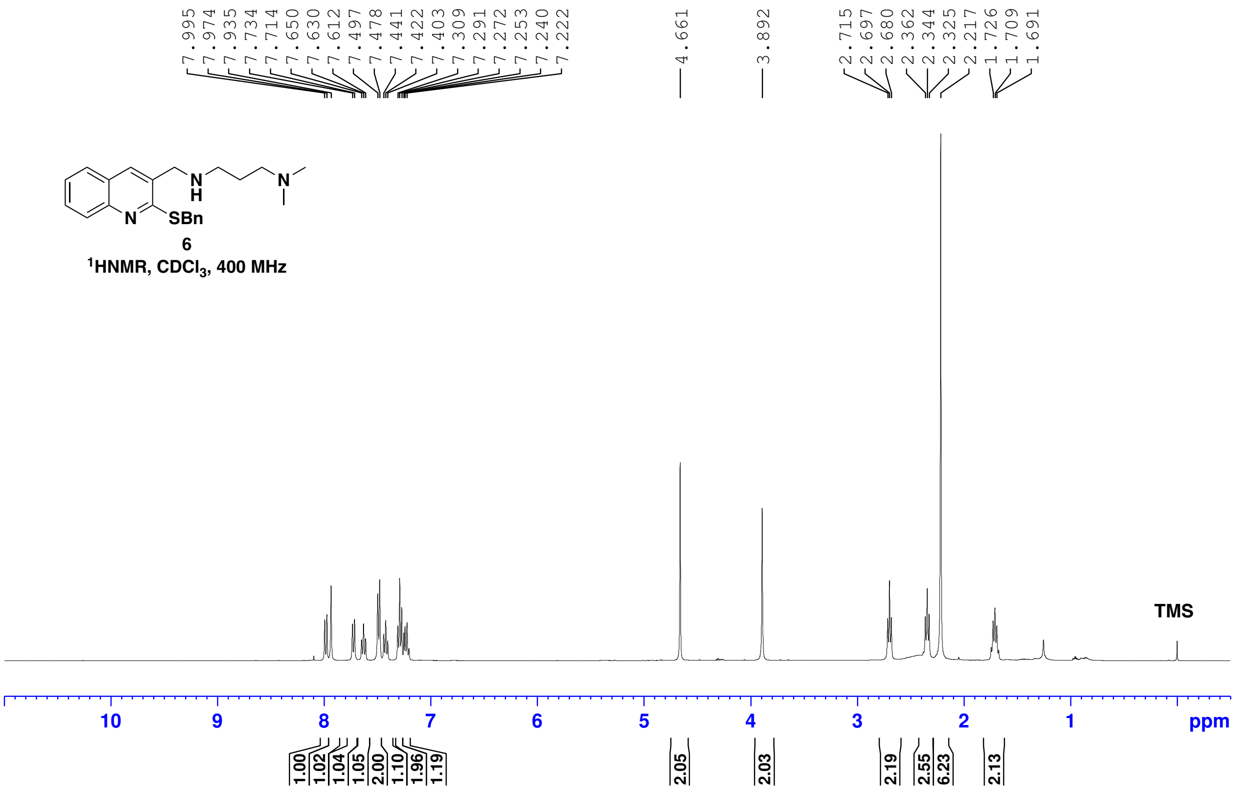
7.995 7.974 7.935 7.734 7.714 7.650 7.630 7.612 7.497 7.478 7.441 7.422 7.403 7.309 7.291 7.272 7.253 7.240 7.222
4.661
3.892
2.715 2.697 2.680 2.362 2.344 2.325 2.217 1.726 1.709 1.691
N
H
N
N
SBn
6
1HNMR, CDCl3, 400 MHz
TMS
10 9 8 7 6 5 4 3 2 1 ppm
1.00 1.02 1.04 1.05 2.00 1.10 1.96 1.19
2.05
2.03
2.19 2.55 6.23 2.13

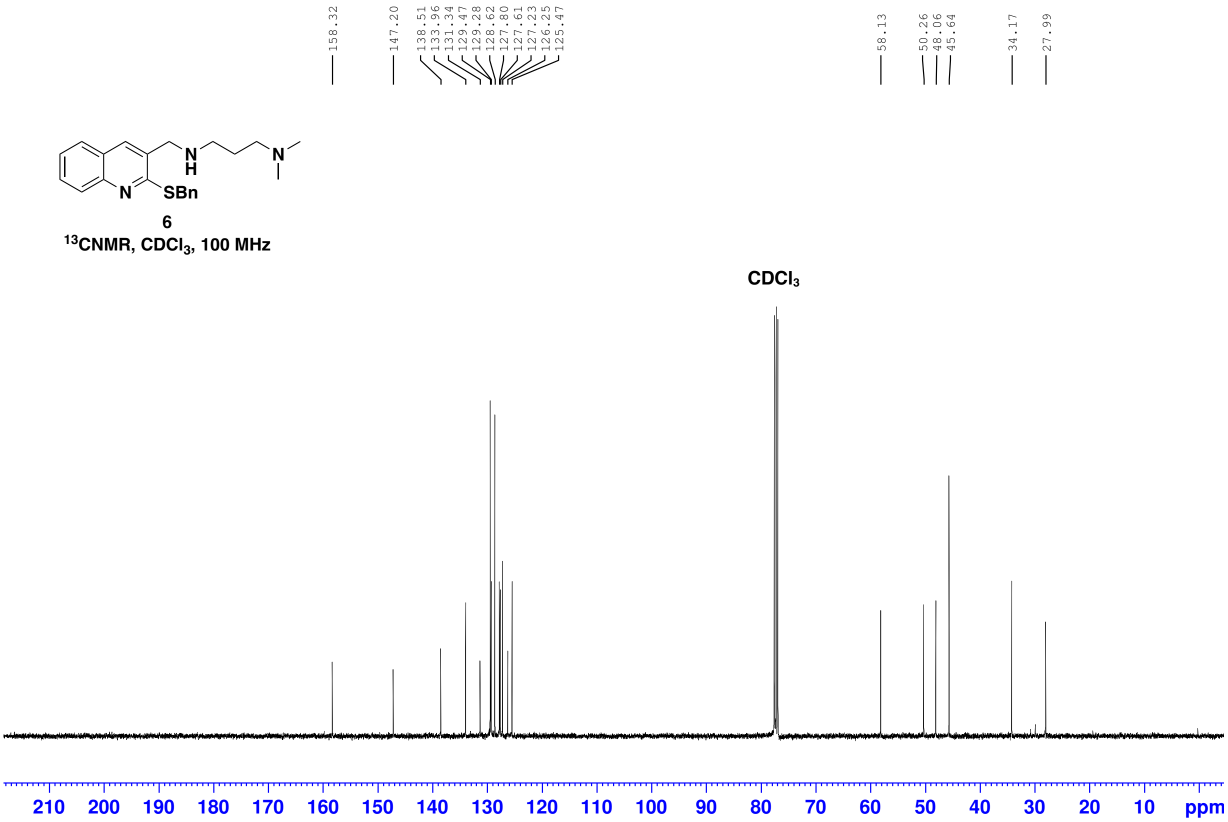
158.32
147.20
138.51 133.96 131.34 129.47 129.28 128.62 127.80 127.61 127.23 126.25 125.47
58.13
50.26 48.06 45.64
34.17
27.99
N
H
N
N
SBn
6
13CNMR, CDCl3, 100 MHz
CDCl3
210 200 190 180 170 160 150 140 130 120 110 100 90 80 70 60 50 40 30 20 10 ppm

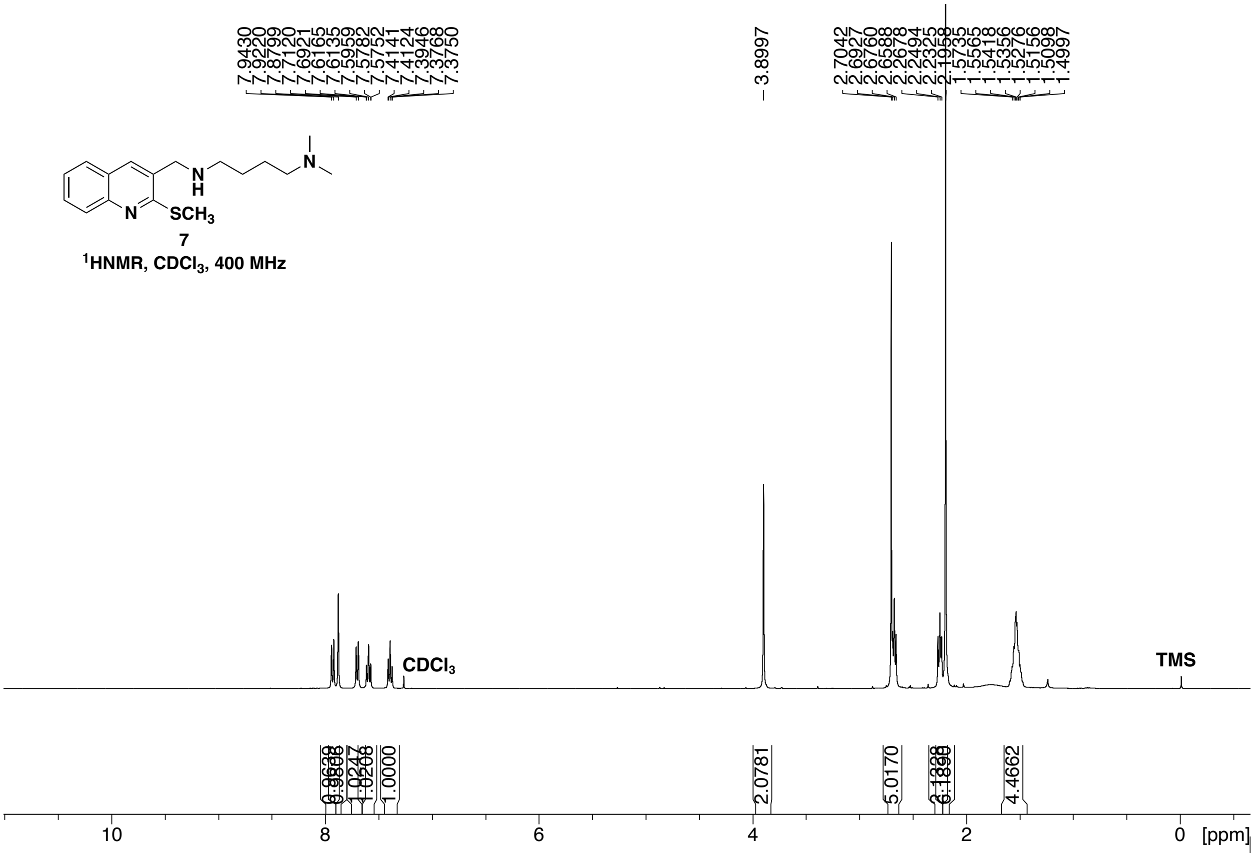
7.9430
7.9220
7.8799
7.7120
7.6921
7.6165
7.6135
7.5959
7.5782
7.5752
7.4141
7.4124
7.3946
7.3768
7.3750
3.8997
2.7042
2.6927
2.6760
2.6588
2.2678
2.2494
2.2325
2.1958
1.5735
1.5565
1.5418
1.5356
1.5276
1.5156
1.5098
1.4997
N
H
N
N
SCH3
7
1HNMR, CDCl3, 400 MHz
CDCl3
TMS
1.0247
1.0208
1.0000
2.0781
5.0170
4.4662
10
8
6
4
2
0
[ppm]

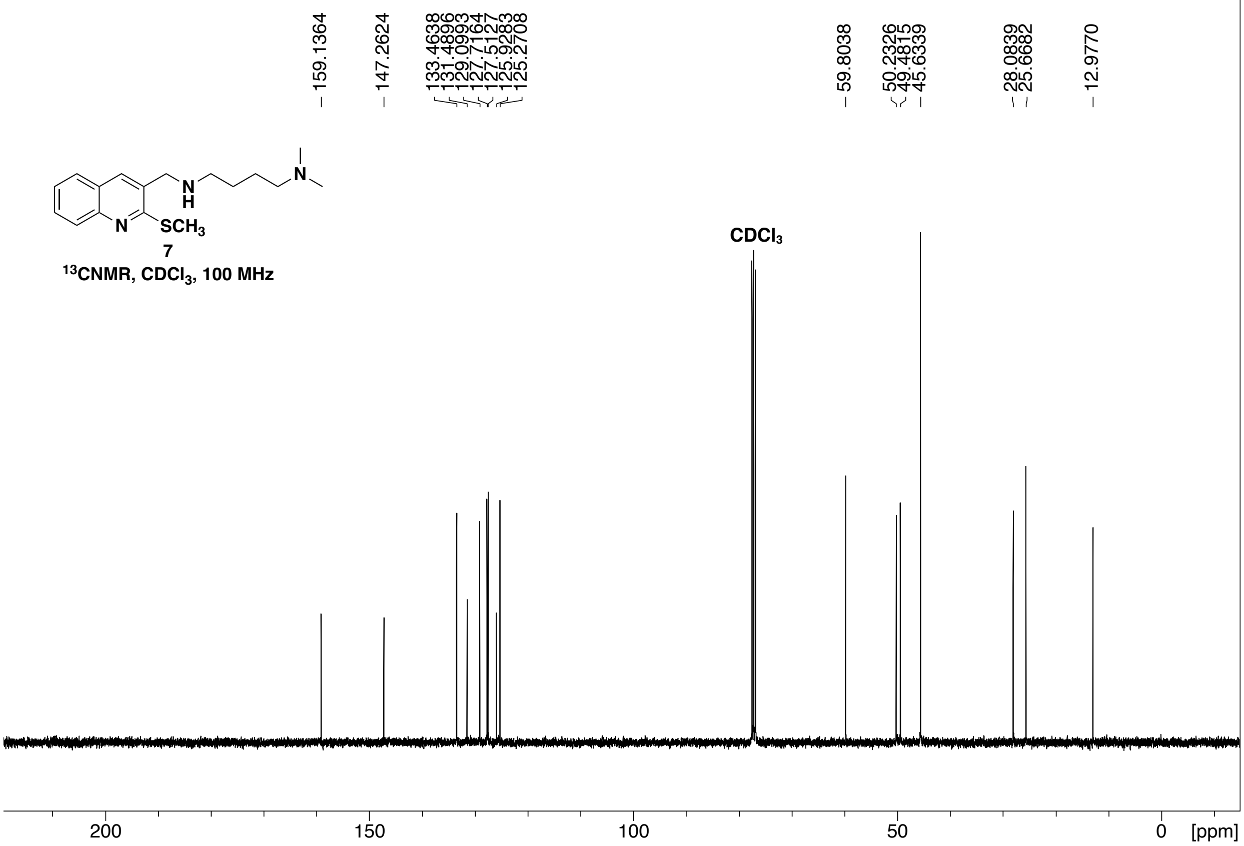
159.1364
147.2624
133.4638
131.4896
129.0993
127.7164
127.5127
125.9283
125.2708
59.8038
50.2326
49.4815
45.6339
28.0839
25.6682
12.9770
N
H
N
N
SCH3
7
13CNMR, CDCl3, 100 MHz
CDCl3
200
150
100
50
0
[ppm]

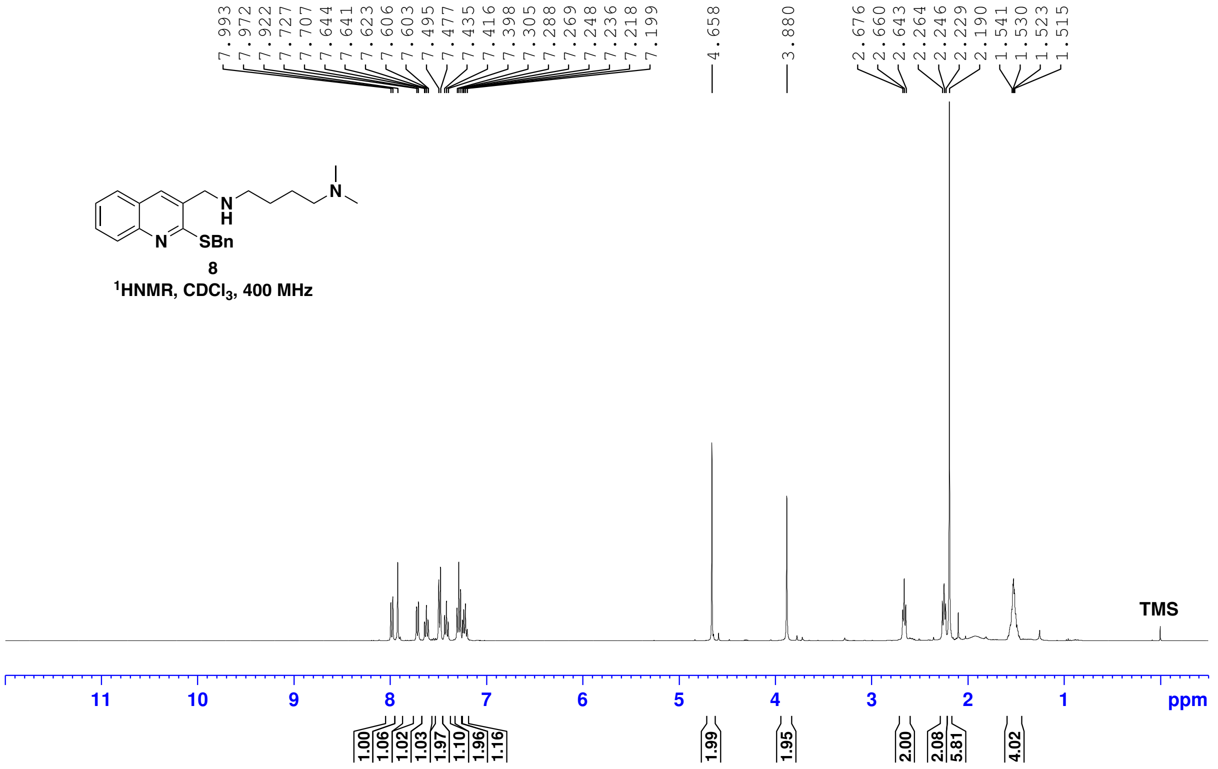
7.993
7.972
7.922
7.727
7.707
7.644
7.641
7.623
7.606
7.603
7.495
7.477
7.435
7.416
7.398
7.305
7.288
7.269
7.248
7.236
7.218
7.199
4.658
3.880
2.676
2.660
2.643
2.264
2.246
2.229
2.190
1.541
1.530
1.523
1.515
N
H
N
N
SBn
8
¹HNMR, CDCl₃, 400 MHz
TMS
11
10
9
8
7
6
5
4
3
2
1
ppm
1.00
1.06
1.02
1.03
1.97
1.10
1.96
1.16
1.99
1.95
2.00
2.08
5.81
4.02

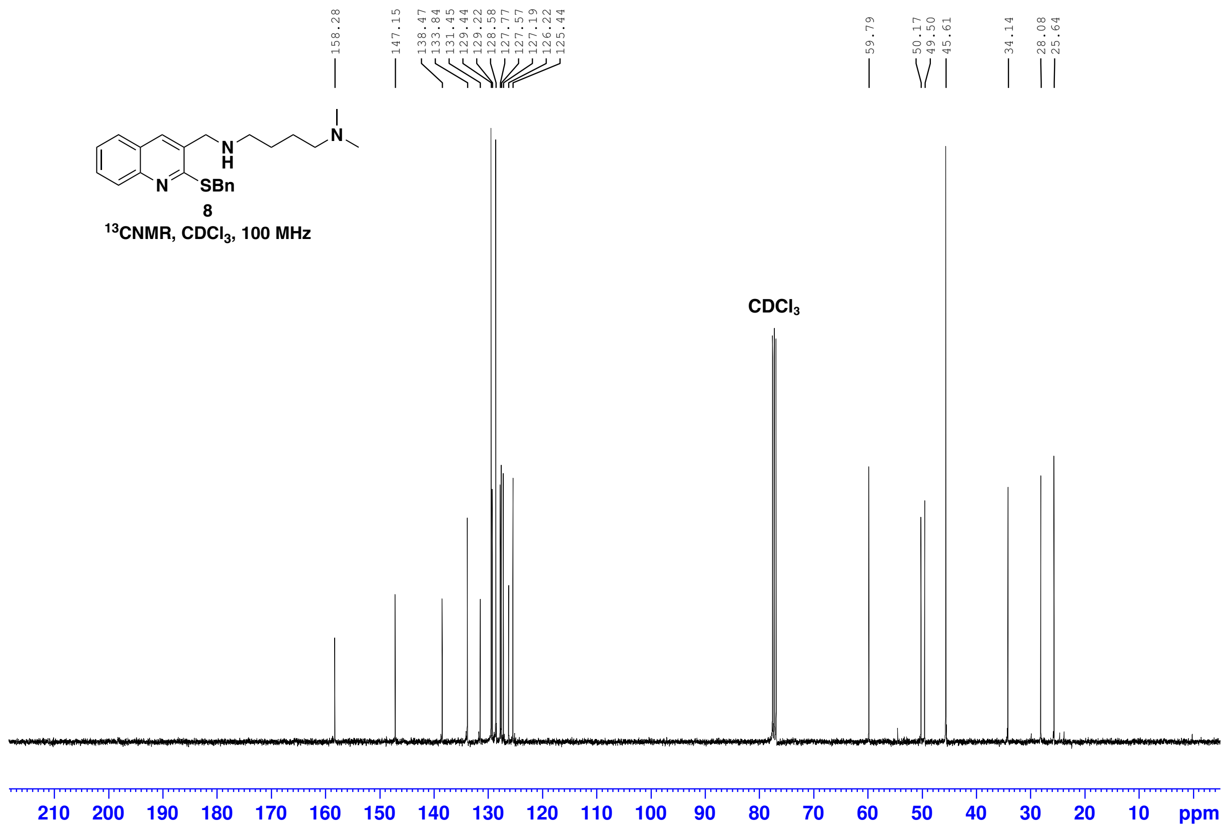
158.28
147.15
138.47
133.84
131.45
129.44
129.22
128.58
127.77
127.57
127.19
126.22
125.44
59.79
50.17
49.50
45.61
34.14
28.08
25.64
N
H
N
N
SBn
8
¹³CNMR, CDCl₃, 100 MHz
CDCl₃
210
200
190
180
170
160
150
140
130
120
110
100
90
80
70
60
50
40
30
20
10
ppm

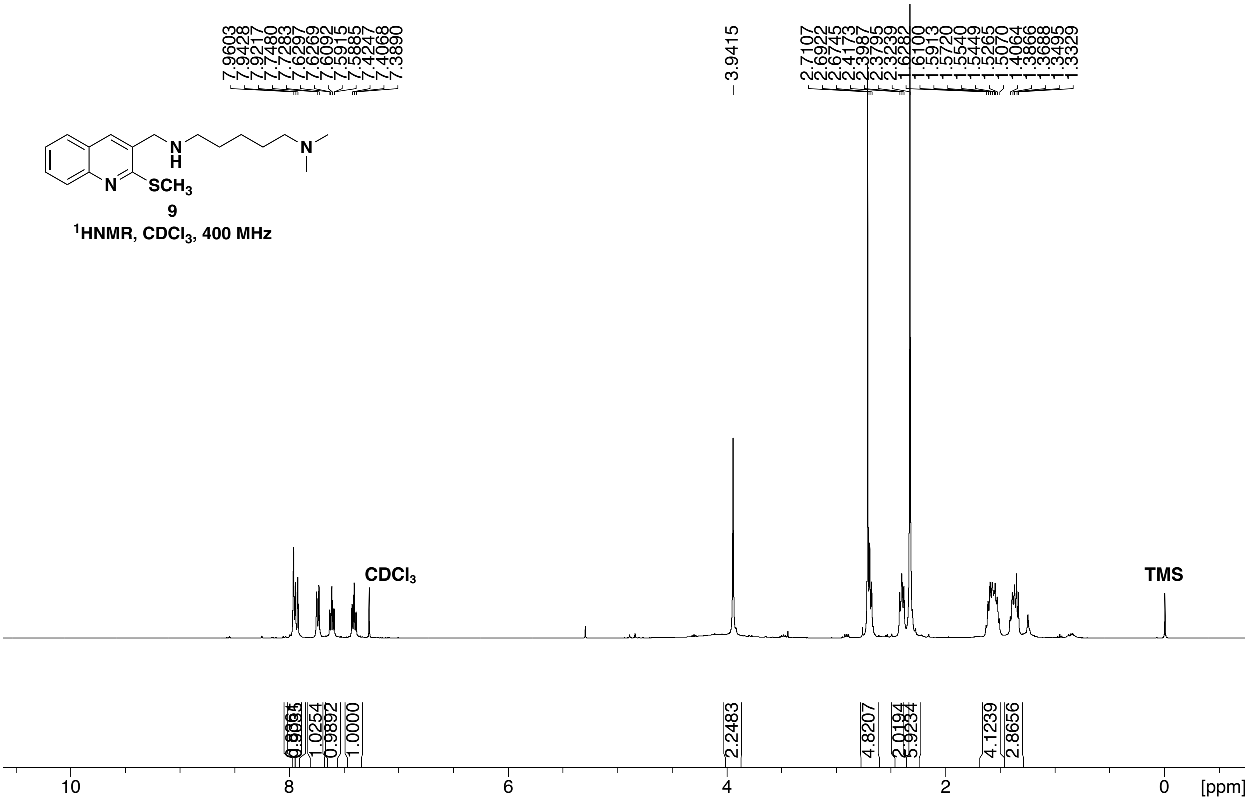
7.9603 7.9428 7.9217 7.7480 7.7283 7.6297 7.6269 7.6092 7.5915 7.5885 7.4247 7.4068 7.3890
3.9415
2.7107 2.6922 2.6743 2.4173 2.3987 2.3795 2.3239 1.6282 1.6100 1.5913 1.5720 1.5540 1.5449 1.5265 1.5070 1.4064 1.3866 1.3688 1.3495 1.3329
N
H
N
SCH3
9
1HNMR, CDCl3, 400 MHz
CDCl3
TMS
1.0254 0.9892 1.0000
2.2483
4.8207 2.0194 5.9234
4.1239 2.8656
10 8 6 4 2 0 [ppm]

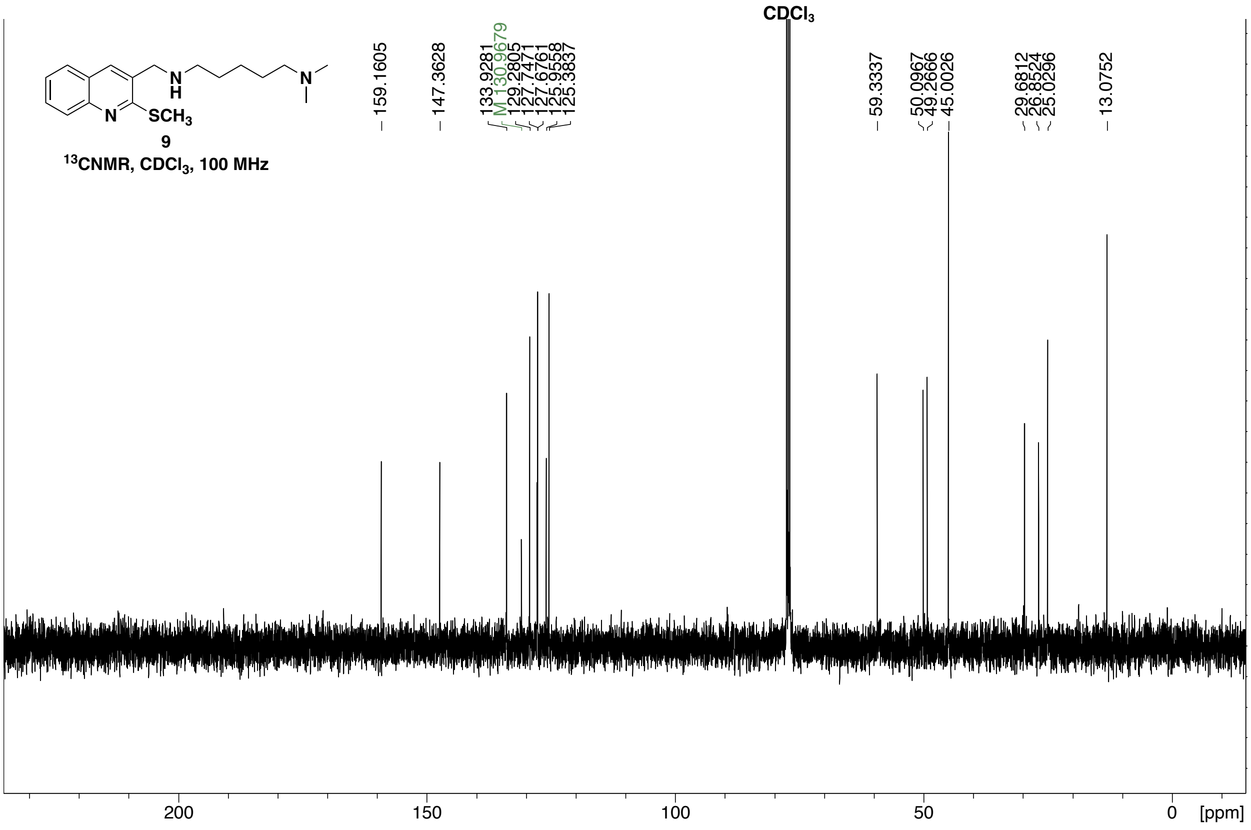
CDCl3
159.1605
147.3628
133.9281 M 130.9679 129.2805 127.7471 127.6761 125.9558 125.3837
59.3337
50.0967 49.2666 45.0026
29.6812 26.8524 25.0296
13.0752
N
H
N
SCH3
9
13CNMR, CDCl3, 100 MHz
200 150 100 50 0 [ppm]

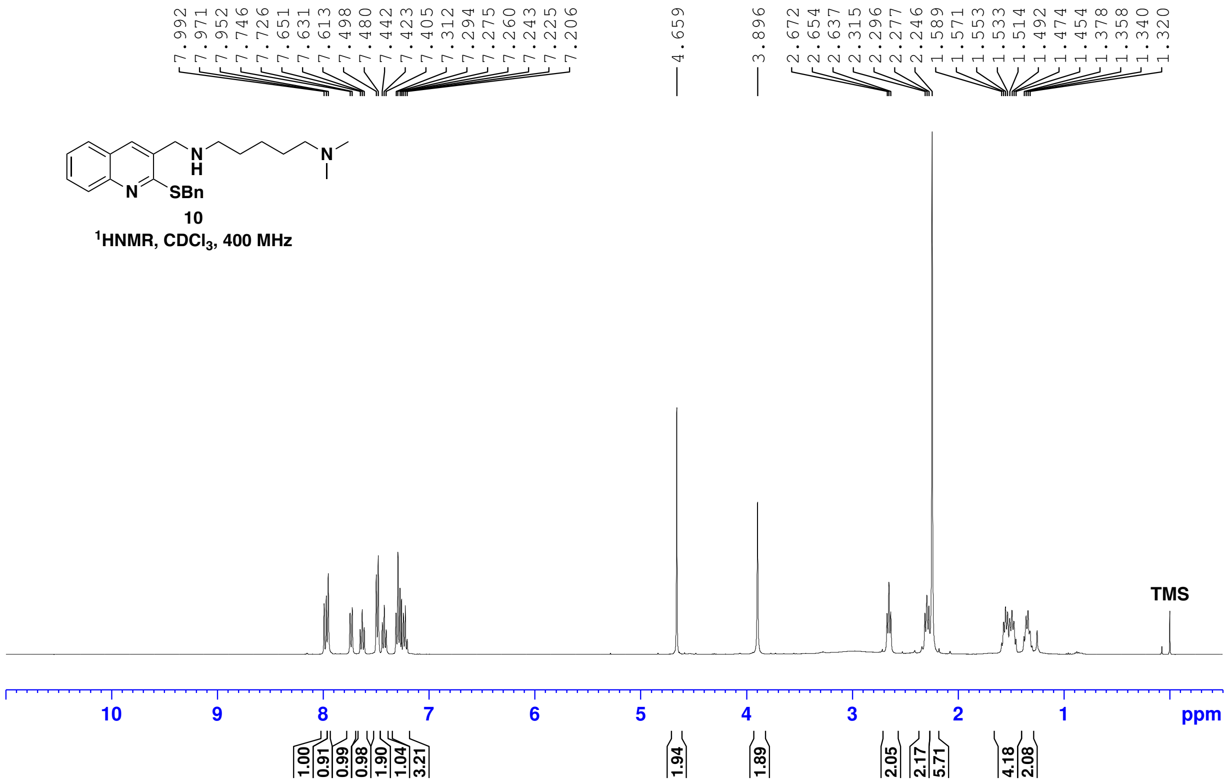
7.992
7.971
7.952
7.746
7.726
7.651
7.631
7.613
7.498
7.480
7.442
7.423
7.405
7.312
7.294
7.275
7.260
7.243
7.225
7.206
4.659
3.896
2.672
2.654
2.637
2.315
2.296
2.277
2.246
1.589
1.571
1.553
1.533
1.514
1.492
1.474
1.454
1.378
1.358
1.340
1.320
N
H
N
SBn
10
$^1$HNMR, CDCl$_3$, 400 MHz
TMS
10 9 8 7 6 5 4 3 2 1 ppm
1.00
0.91
0.99
0.98
1.90
1.04
3.21
1.94
1.89
2.05
2.17
5.71
4.18
2.08

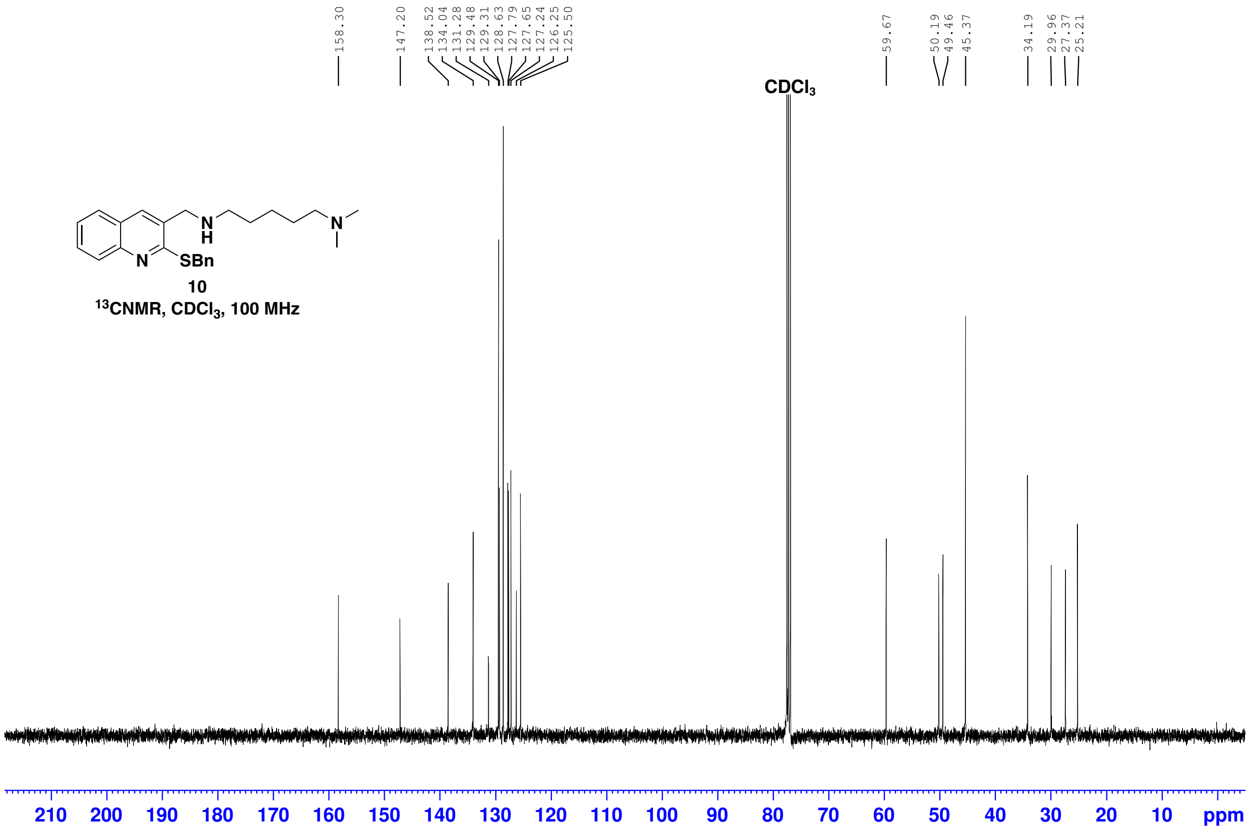
158.30
147.20
138.52
134.04
131.28
129.48
129.31
128.63
127.79
127.65
127.24
126.25
125.50
59.67
50.19
49.46
45.37
34.19
29.96
27.37
25.21
CDCl$_3$
N
H
N
SBn
10
$^{13}$CNMR, CDCl$_3$, 100 MHz
210 200 190 180 170 160 150 140 130 120 110 100 90 80 70 60 50 40 30 20 10 ppm

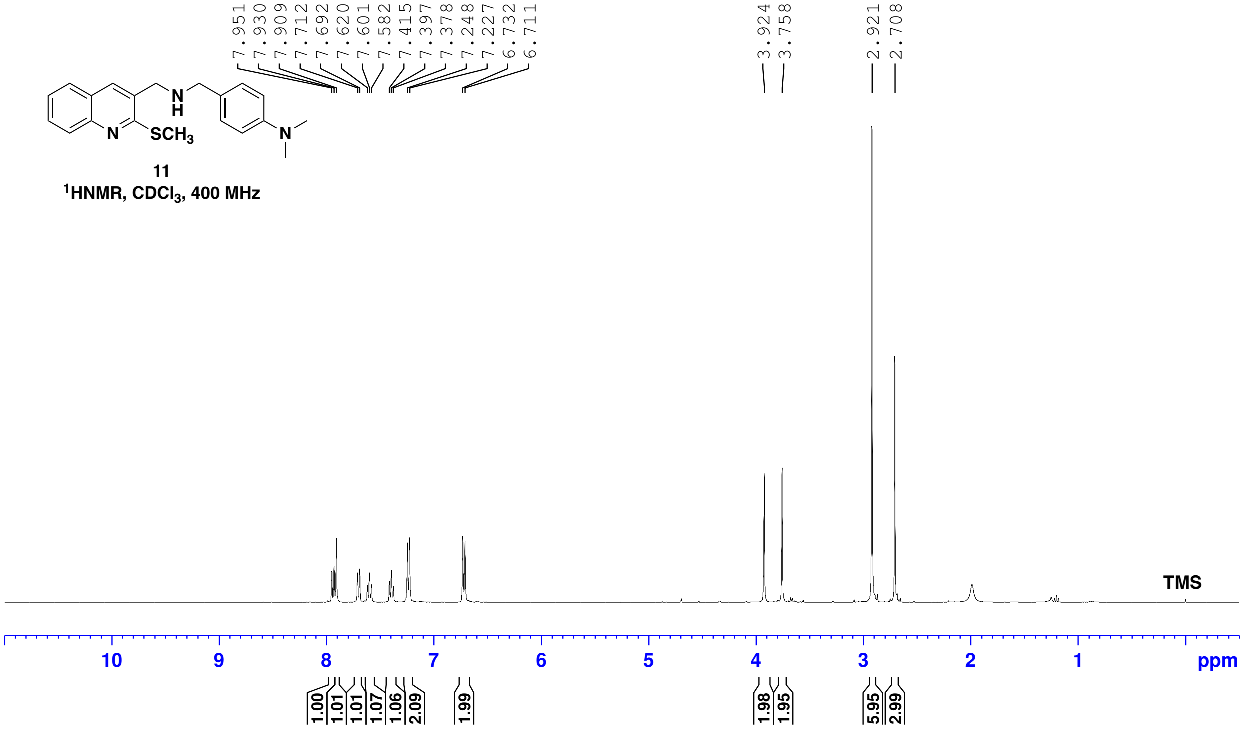


11
$^{1}$HNMR, $CDCl_3$, 400 MHz

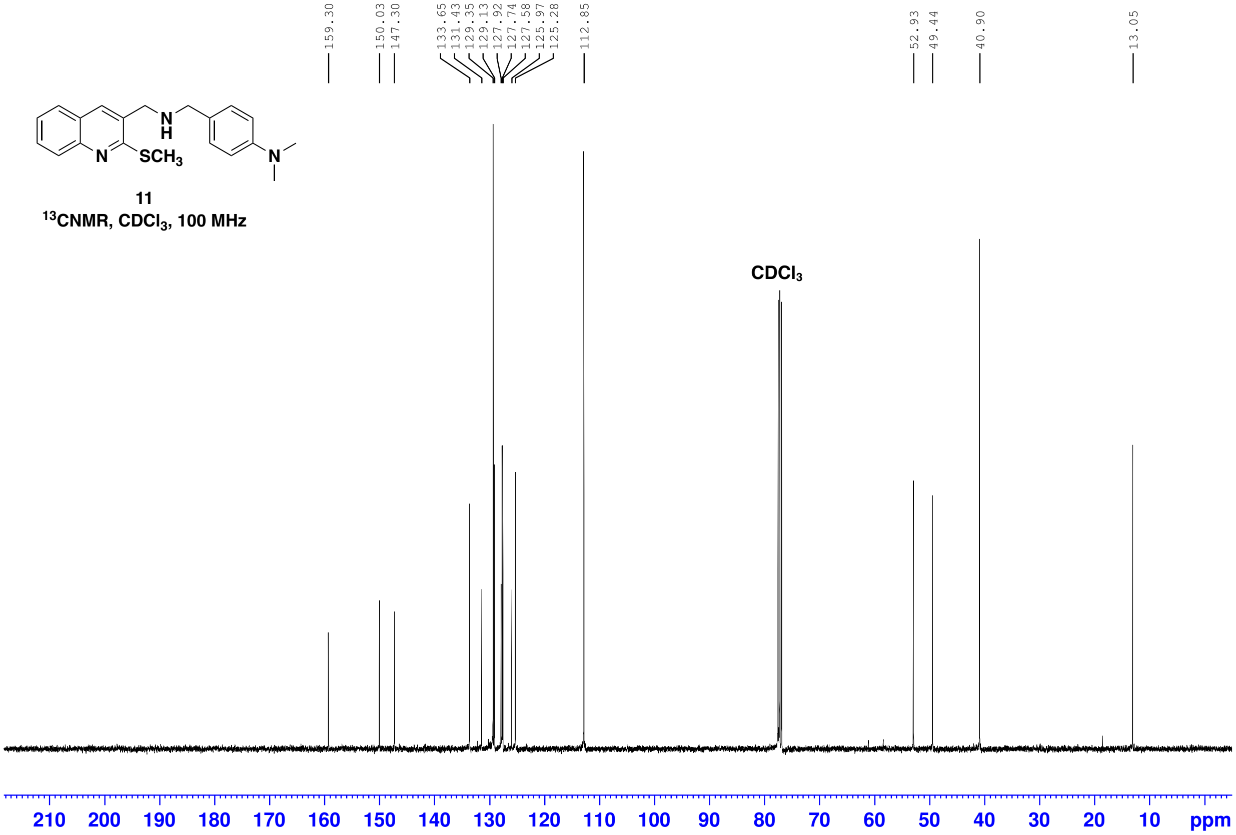


11
$^{13}$CNMR, $CDCl_3$, 100 MHz

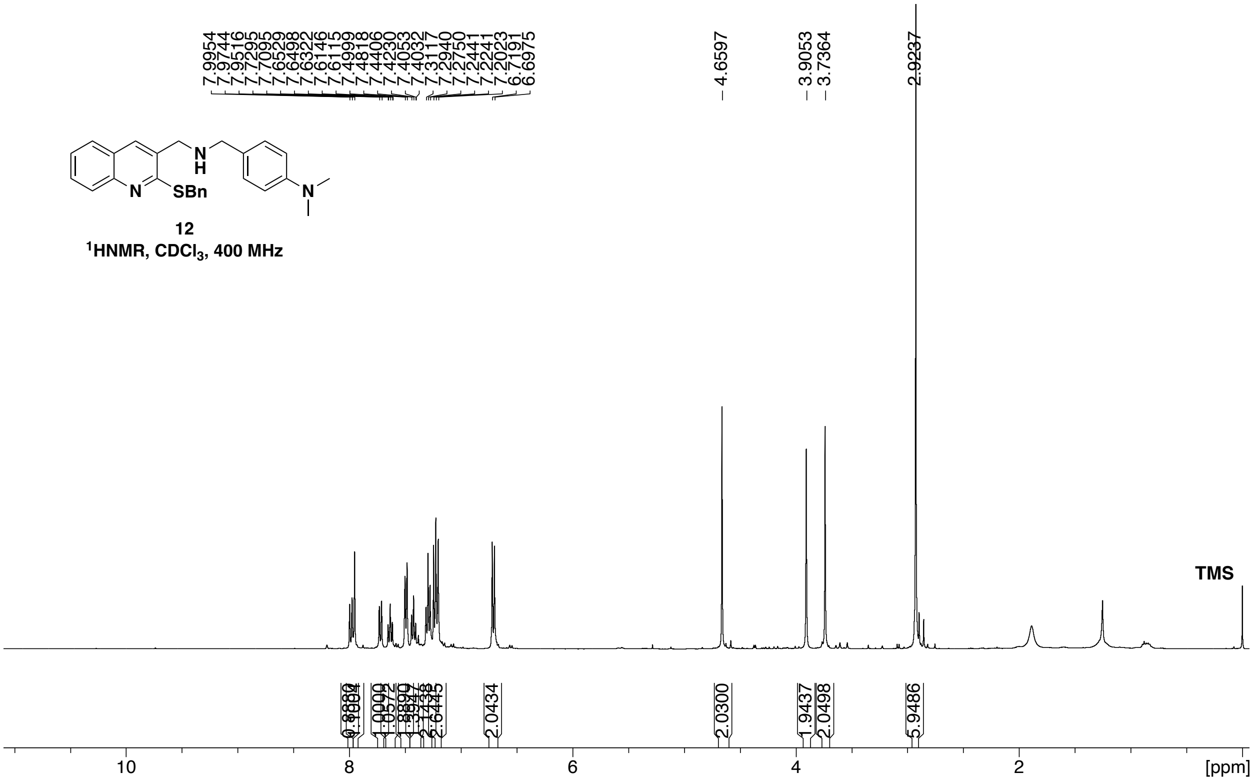


12

$^1$HNMR, $CDCl_3$, 400 MHz

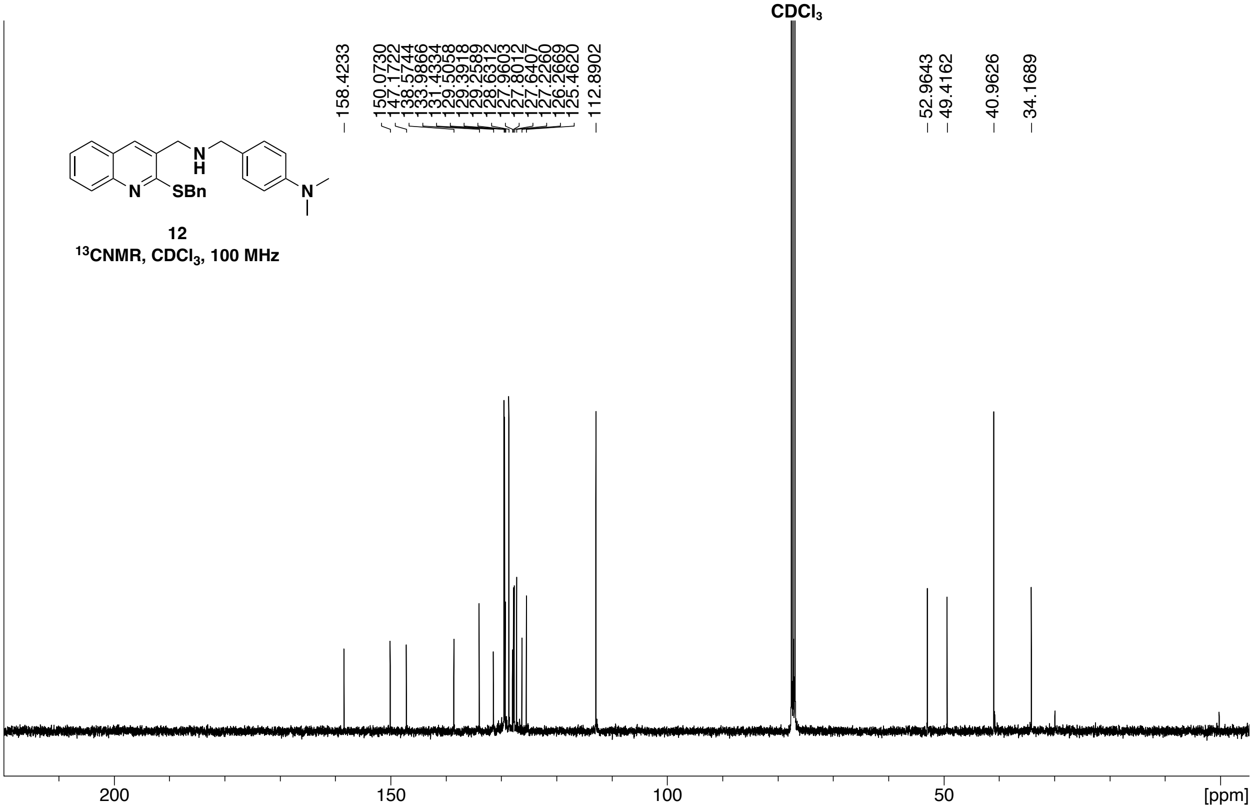


12

$^{13}$CNMR, $CDCl_3$, 100 MHz

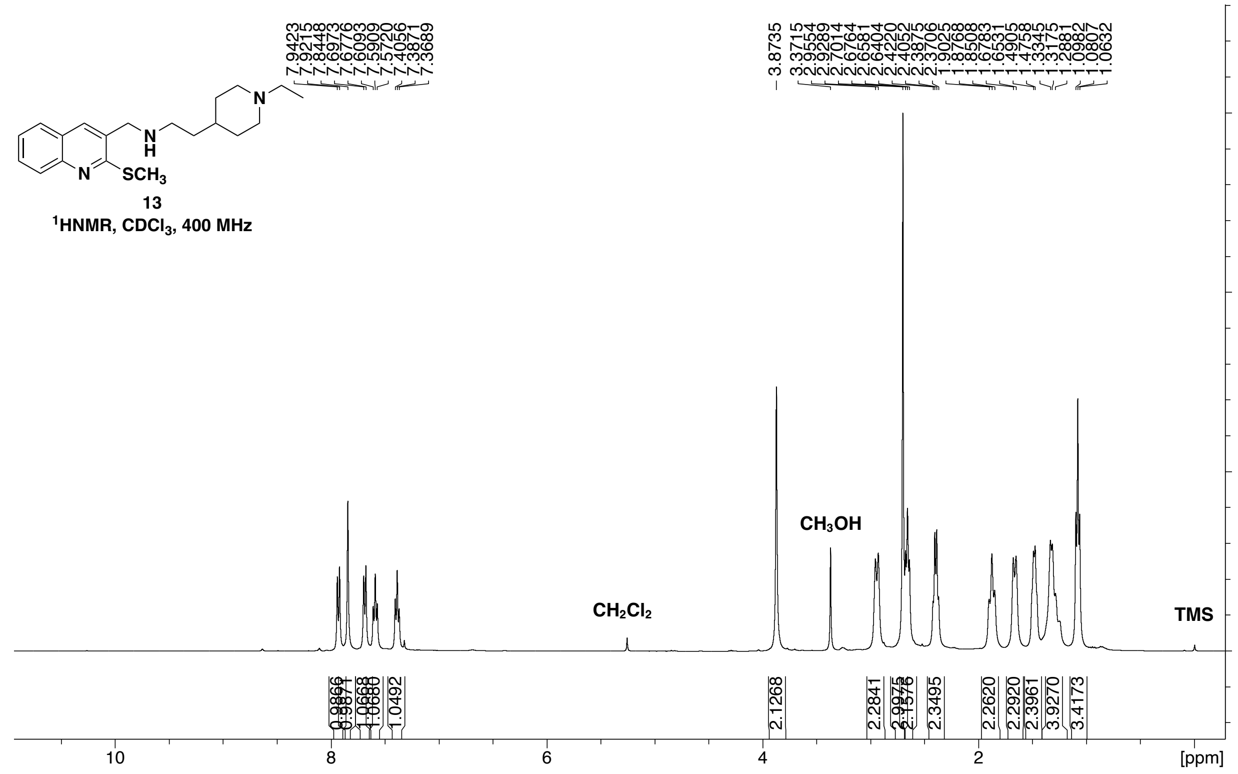
7.9423
7.9215
7.8448
7.6973
7.6776
7.6093
7.5909
7.5720
7.4056
7.3871
7.3689
3.8735
3.3715
3.3554
2.9289
2.7014
2.6764
2.6581
2.6404
2.4220
2.4052
2.3875
2.3706
1.9025
1.8768
1.8508
1.6783
1.6531
1.4905
1.4758
1.3345
1.3175
1.2881
1.0982
1.0807
1.0632
13
$^1$HNMR, CDCl$_3$, 400 MHz
CH$_3$OH
CH$_2$Cl$_2$
TMS
0.9866
0.9871
1.0668
1.0680
1.0492
2.1268
2.2841
2.9975
2.1576
2.3495
2.2620
2.2920
2.3961
3.9270
3.4173
10
8
6
4
2
0
[ppm]

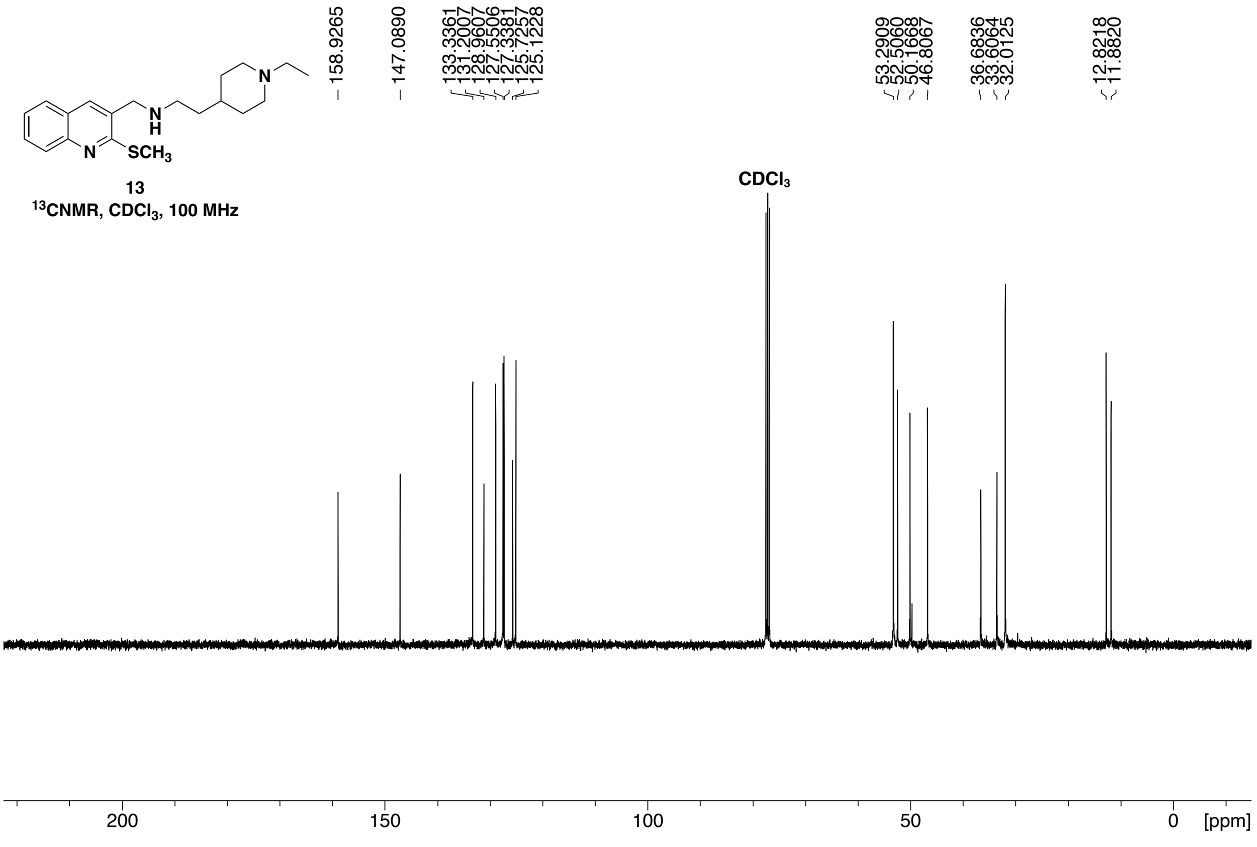
158.9265
147.0890
133.3361
131.2007
128.9607
127.5506
127.3381
125.7257
125.1228
53.2909
52.5060
50.1668
46.8067
36.6836
33.6064
32.0125
12.8218
11.8820
13
$^{13}$CNMR, CDCl$_3$, 100 MHz
CDCl$_3$
200
150
100
50
0
[ppm]

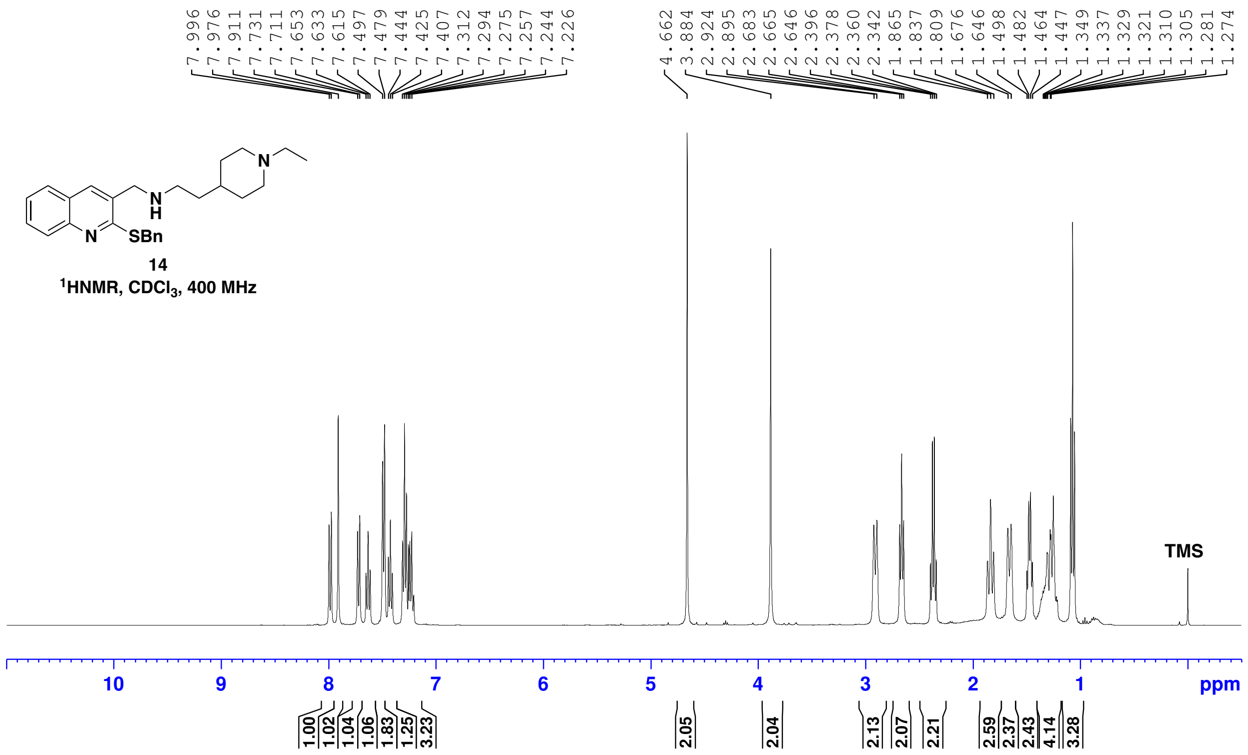
14
$^1$HNMR, CDCl$_3$, 400 MHz
TMS
ppm

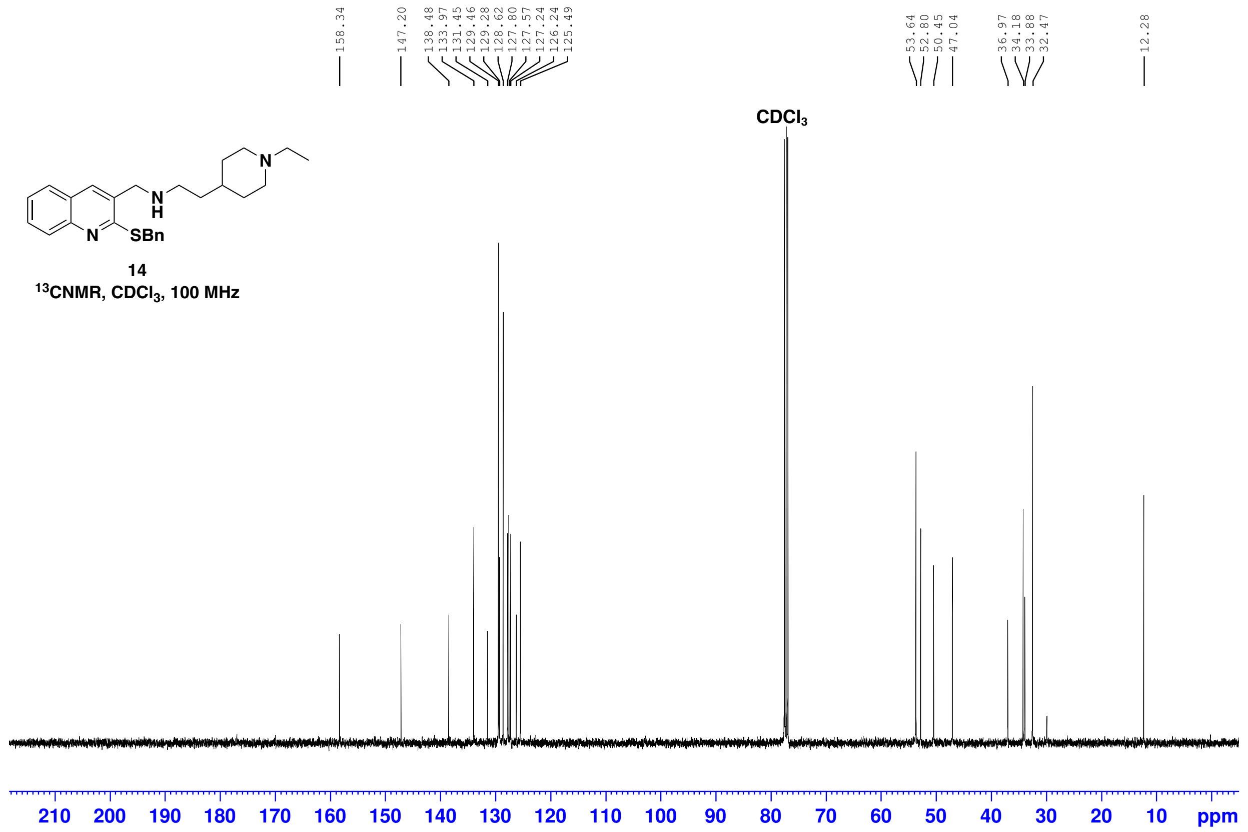
14
$^{13}$CNMR, CDCl$_3$, 100 MHz
CDCl$_3$
ppm

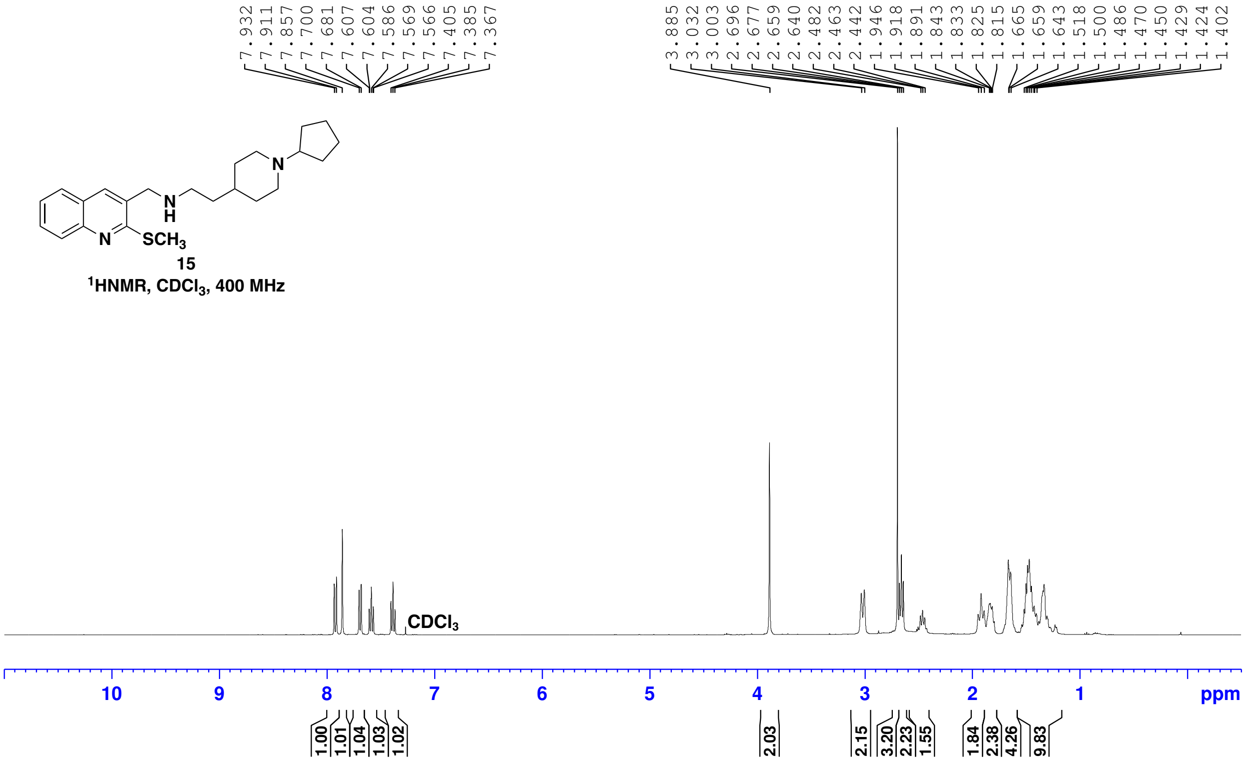
7.932 7.911 7.857 7.700 7.681 7.607 7.604 7.586 7.569 7.566 7.405 7.385 7.367
3.885 3.032 3.003 2.696 2.677 2.659 2.640 2.482 2.463 2.442 1.946 1.918 1.891 1.843 1.833 1.825 1.815 1.665 1.659 1.643 1.518 1.500 1.486 1.470 1.450 1.429 1.424 1.402
N
H
N
N
SCH3
15
1HNMR, CDCl3, 400 MHz
CDCl3
10 9 8 7 6 5 4 3 2 1 ppm
1.00 1.01 1.04 1.03 1.02 2.03 2.15 3.20 2.23 1.55 1.84 2.38 4.26 9.83

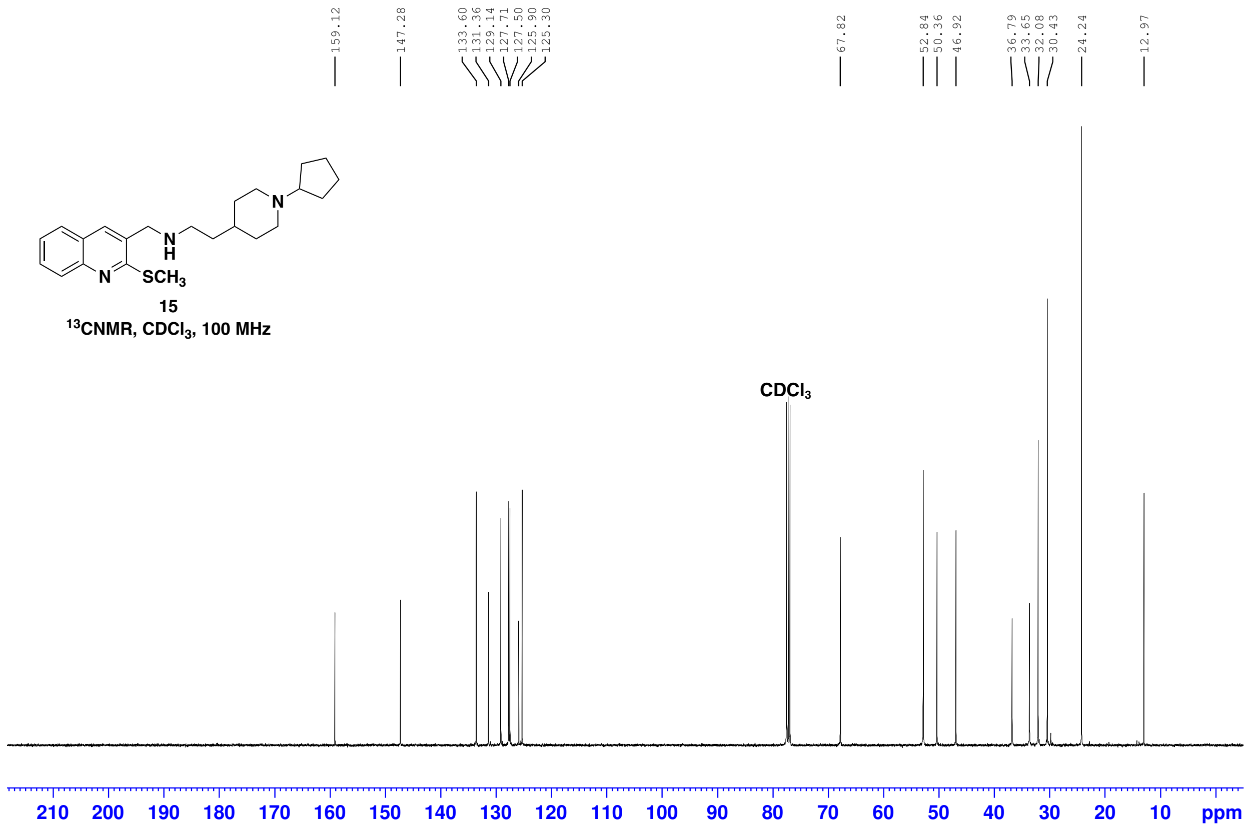
159.12 147.28 133.60 131.36 129.14 127.71 127.50 125.90 125.30 67.82 52.84 50.36 46.92 36.79 33.65 32.08 30.43 24.24 12.97
N
H
N
N
SCH3
15
13CNMR, CDCl3, 100 MHz
CDCl3
210 200 190 180 170 160 150 140 130 120 110 100 90 80 70 60 50 40 30 20 10 ppm

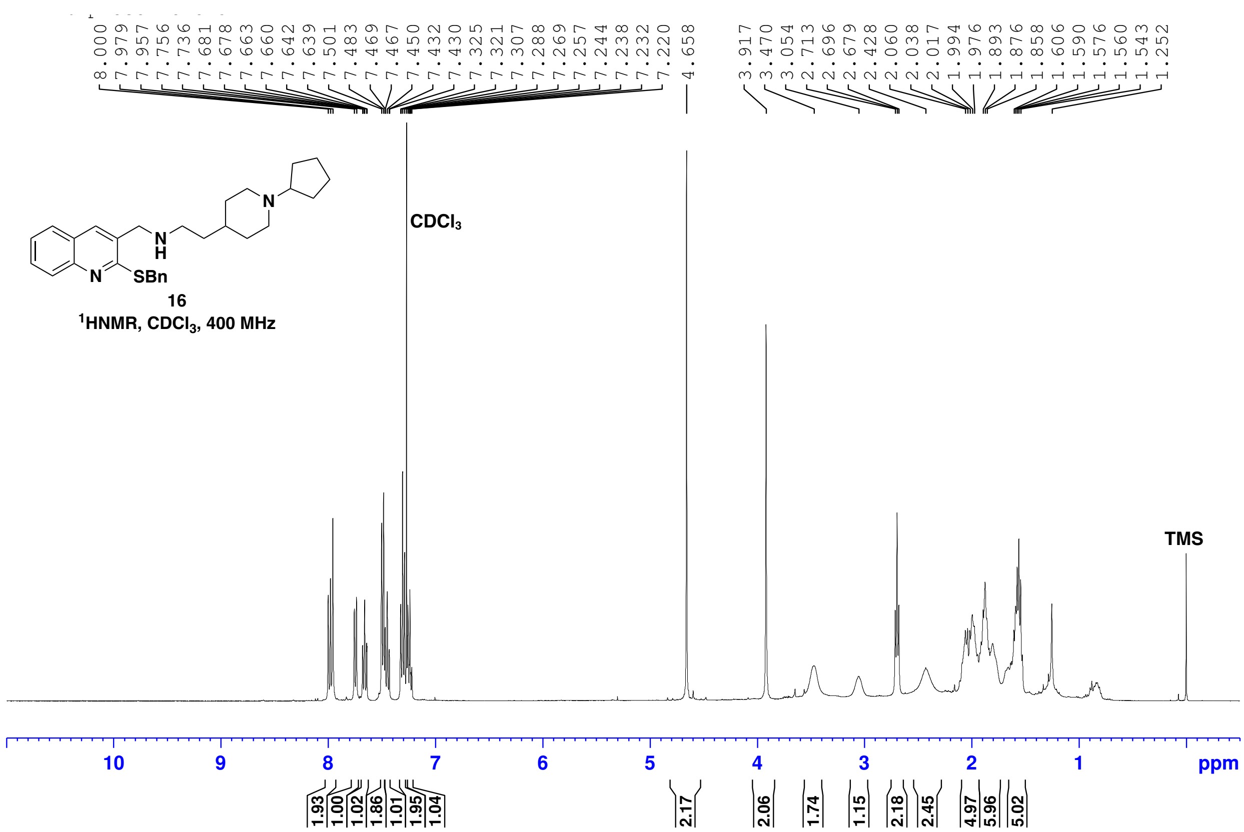
8.000 7.979 7.957 7.756 7.736 7.681 7.678 7.663 7.660 7.642 7.639 7.501 7.483 7.469 7.467 7.450 7.432 7.430 7.325 7.321 7.307 7.288 7.269 7.257 7.244 7.238 7.232 7.220 4.658 3.917 3.470 3.054 2.713 2.696 2.679 2.428 2.060 2.038 2.017 1.994 1.976 1.893 1.876 1.858 1.606 1.590 1.576 1.560 1.543 1.252
N
H
N
N
SBn
16
1HNMR, CDCl3, 400 MHz
CDCl3
TMS
10 9 8 7 6 5 4 3 2 1 ppm
1.93 1.00 1.02 1.86 1.01 1.95 1.04 2.17 2.06 1.74 1.15 2.18 2.45 4.97 5.96 5.02

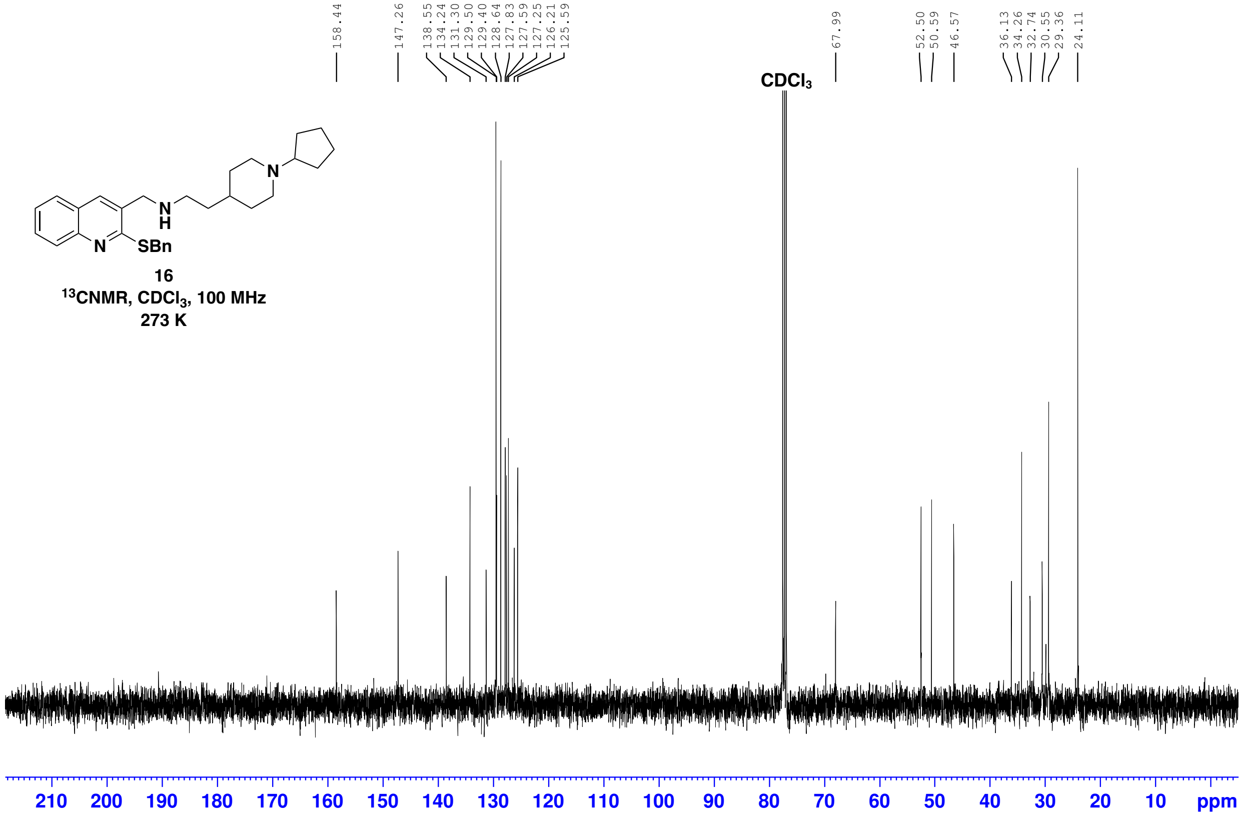
158.44 147.26 138.55 134.24 131.30 129.50 129.40 128.64 127.83 127.59 127.25 126.21 125.59 67.99 52.50 50.59 46.57 36.13 34.26 32.74 30.55 29.36 24.11
CDCl3
N
H
N
N
SBn
16
13CNMR, CDCl3, 100 MHz
273 K
210 200 190 180 170 160 150 140 130 120 110 100 90 80 70 60 50 40 30 20 10 ppm

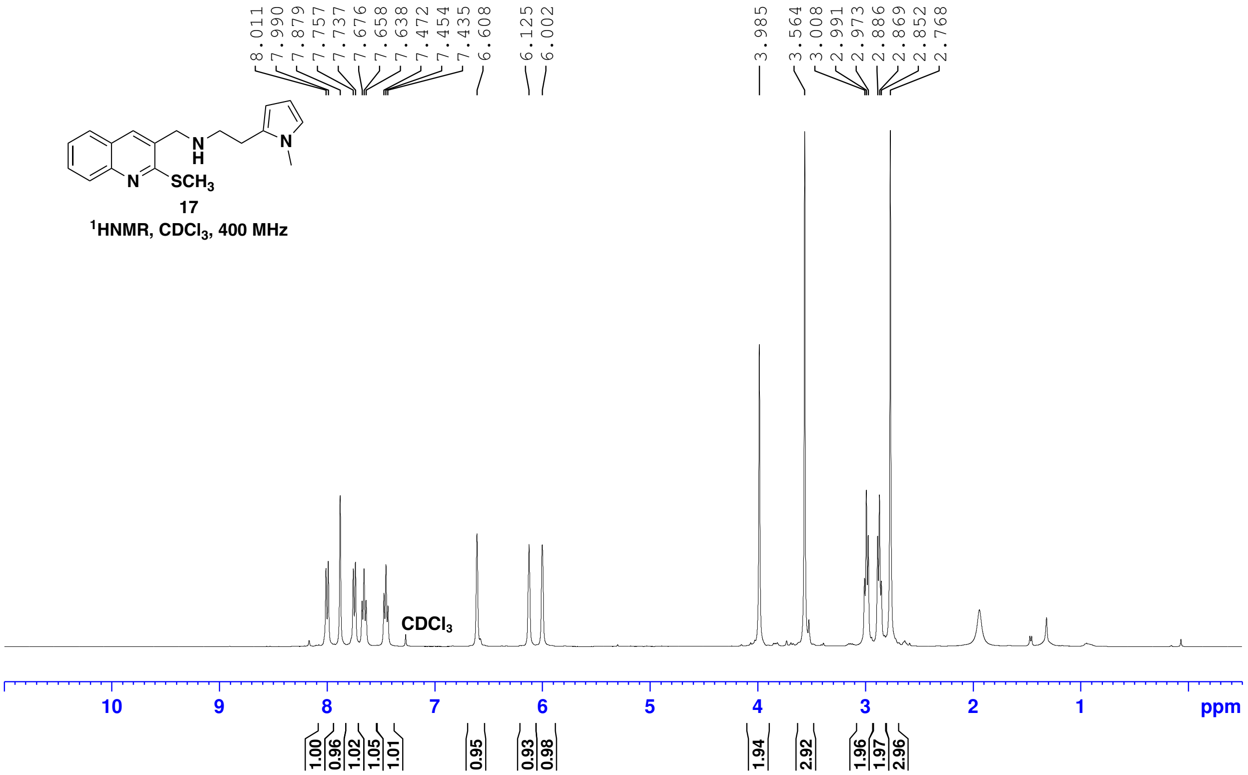

8.011
7.990
7.879
7.757
7.737
7.676
7.658
7.638
7.472
7.454
7.435
6.608
6.125
6.002
3.985
3.564
3.008
2.991
2.973
2.886
2.869
2.852
2.768
N
H
N
N
SCH3
17
1HNMR, CDCl3, 400 MHz
CDCl3
10
9
8
7
6
5
4
3
2
1
ppm
1.00
0.96
1.02
1.05
1.01
0.95
0.93
0.98
1.94
2.92
1.96
1.97
2.96


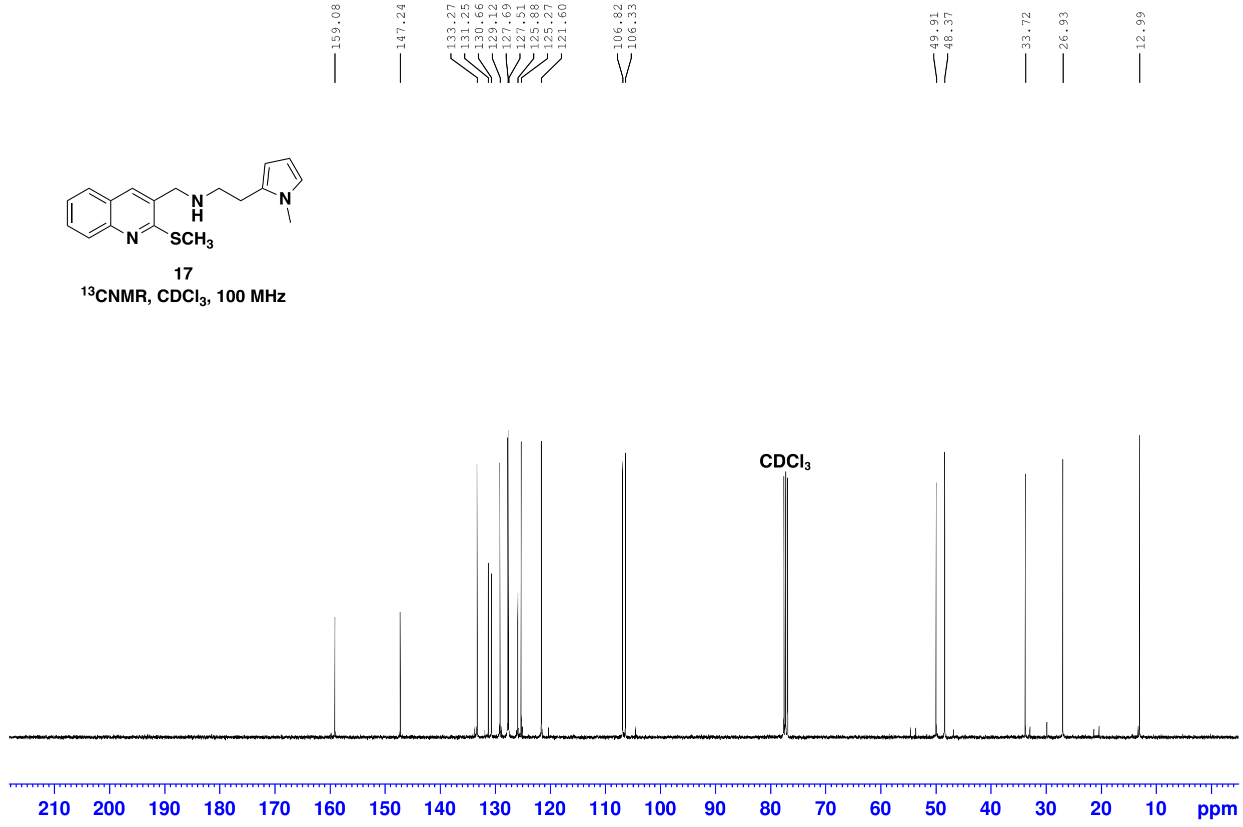

159.08
147.24
133.27
131.25
130.66
129.12
127.69
127.51
125.88
125.27
121.60
106.82
106.33
49.91
48.37
33.72
26.93
12.99
N
H
N
N
SCH3
17
13CNMR, CDCl3, 100 MHz
CDCl3
210
200
190
180
170
160
150
140
130
120
110
100
90
80
70
60
50
40
30
20
10
ppm

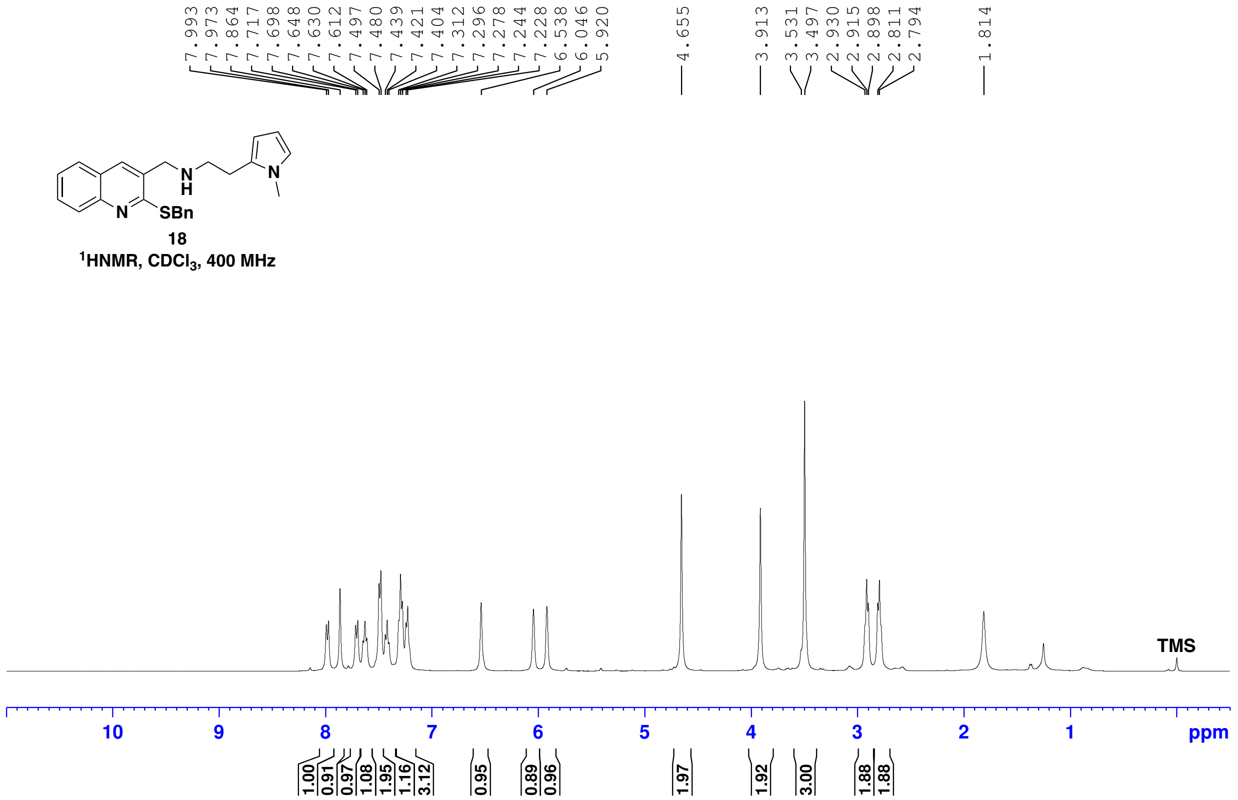
7.993
7.973
7.864
7.717
7.698
7.648
7.630
7.612
7.497
7.480
7.439
7.421
7.404
7.312
7.296
7.278
7.244
7.228
6.538
6.046
5.920
4.655
3.913
3.531
3.497
2.930
2.915
2.898
2.811
2.794
1.814
N
H
N
SBn
N
18
1HNMR, CDCl3, 400 MHz
TMS
10
9
8
7
6
5
4
3
2
1
ppm
1.00
0.91
0.97
1.08
1.95
1.16
3.12
0.95
0.89
0.96
1.97
1.92
3.00
1.88
1.88

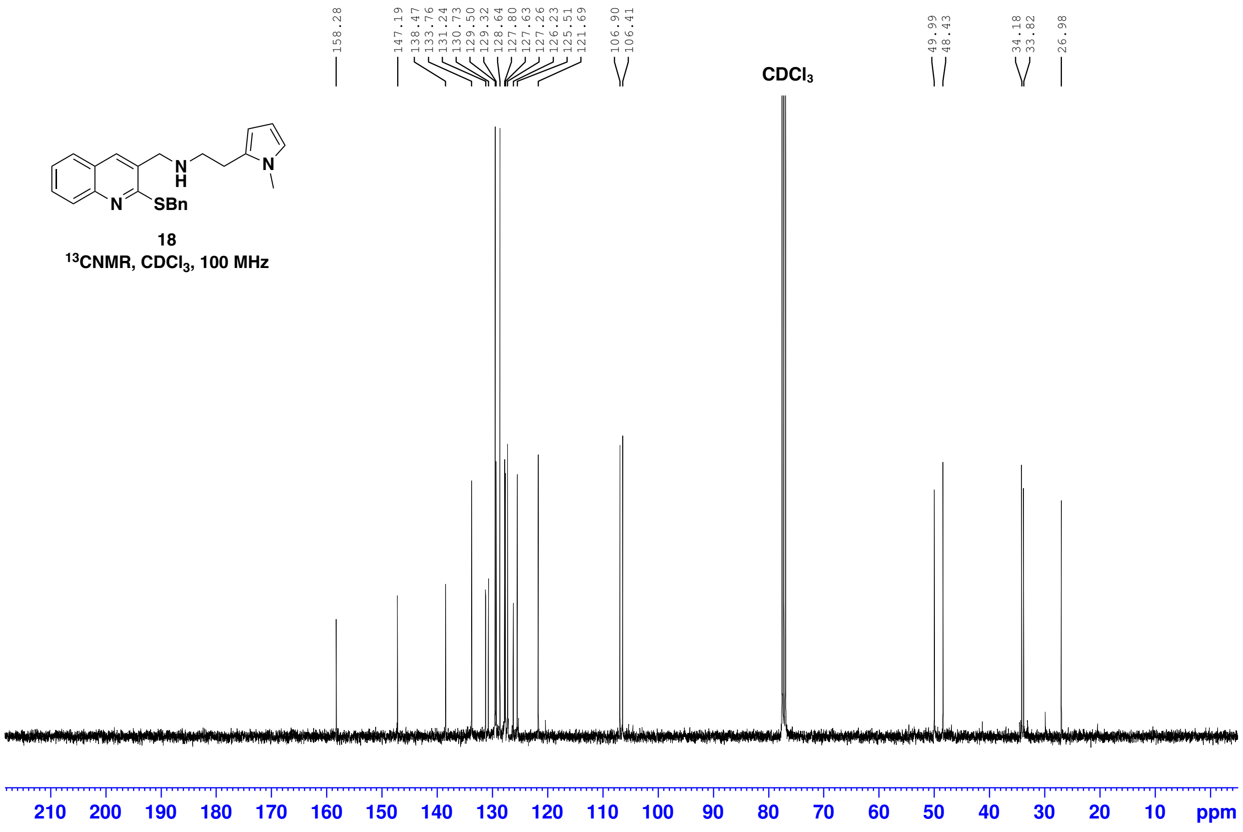
158.28
147.19
138.47
133.76
131.24
130.73
129.50
129.32
128.64
127.80
127.63
127.26
126.23
125.51
121.69
106.90
106.41
CDCl3
49.99
48.43
34.18
33.82
26.98
N
H
N
SBn
N
18
13CNMR, CDCl3, 100 MHz
210
200
190
180
170
160
150
140
130
120
110
100
90
80
70
60
50
40
30
20
10
ppm

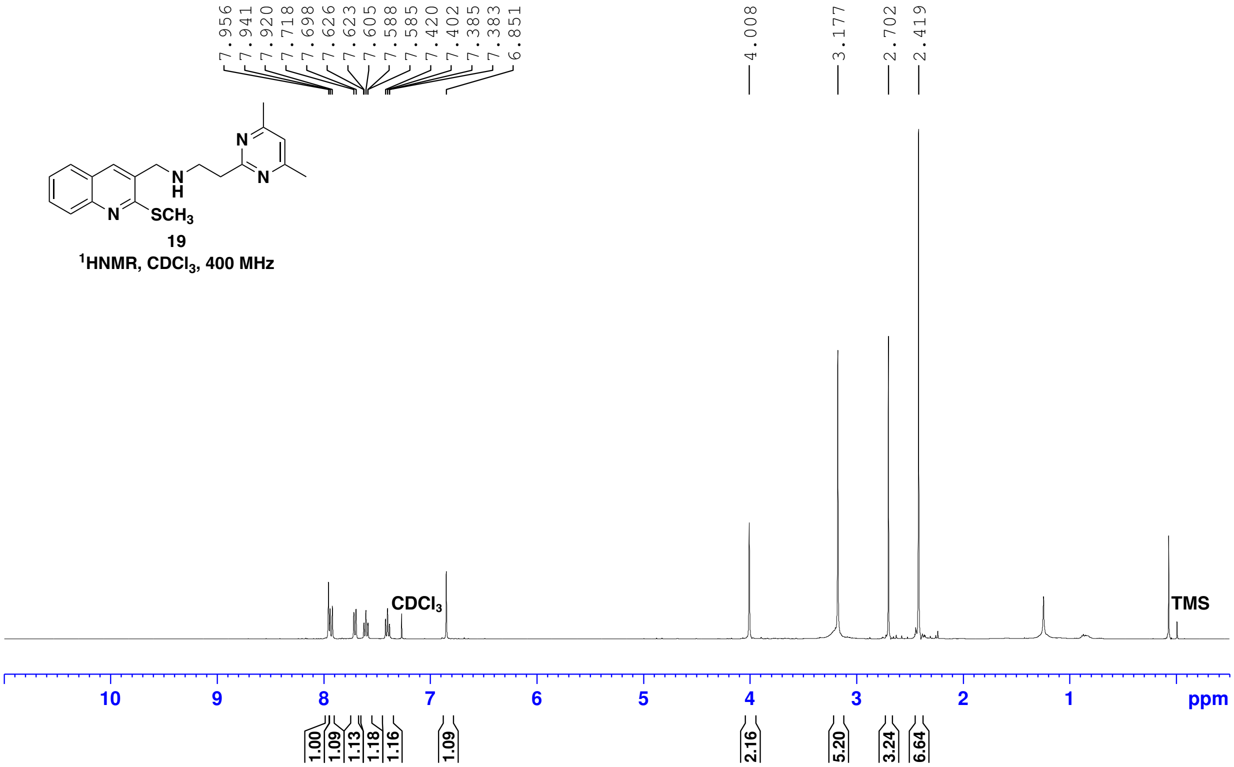
7.956 7.941 7.920 7.718 7.698 7.626 7.623 7.605 7.588 7.585 7.420 7.402 7.385 7.383 6.851
4.008
3.177
2.702
2.419
N
N
N
H
N
SCH3
19
1HNMR, CDCl3, 400 MHz
CDCl3
TMS
10 9 8 7 6 5 4 3 2 1 ppm
1.00 1.09 1.13 1.18 1.16 1.09 2.16 5.20 3.24 6.64

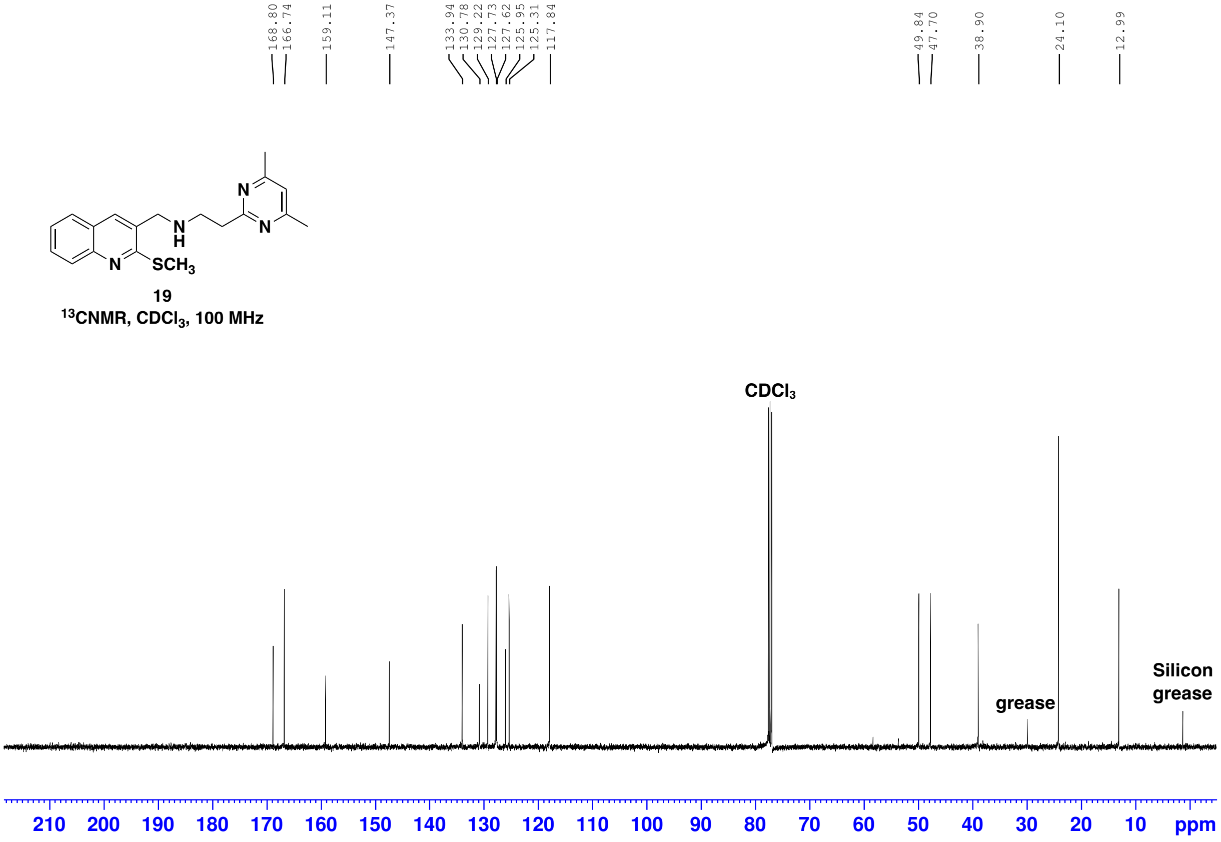
168.80 166.74 159.11 147.37 133.94 130.78 129.22 127.73 127.62 125.95 125.31 117.84 49.84 47.70 38.90 24.10 12.99
N
N
N
H
N
SCH3
19
13CNMR, CDCl3, 100 MHz
CDCl3
grease
Silicon grease
210 200 190 180 170 160 150 140 130 120 110 100 90 80 70 60 50 40 30 20 10 ppm

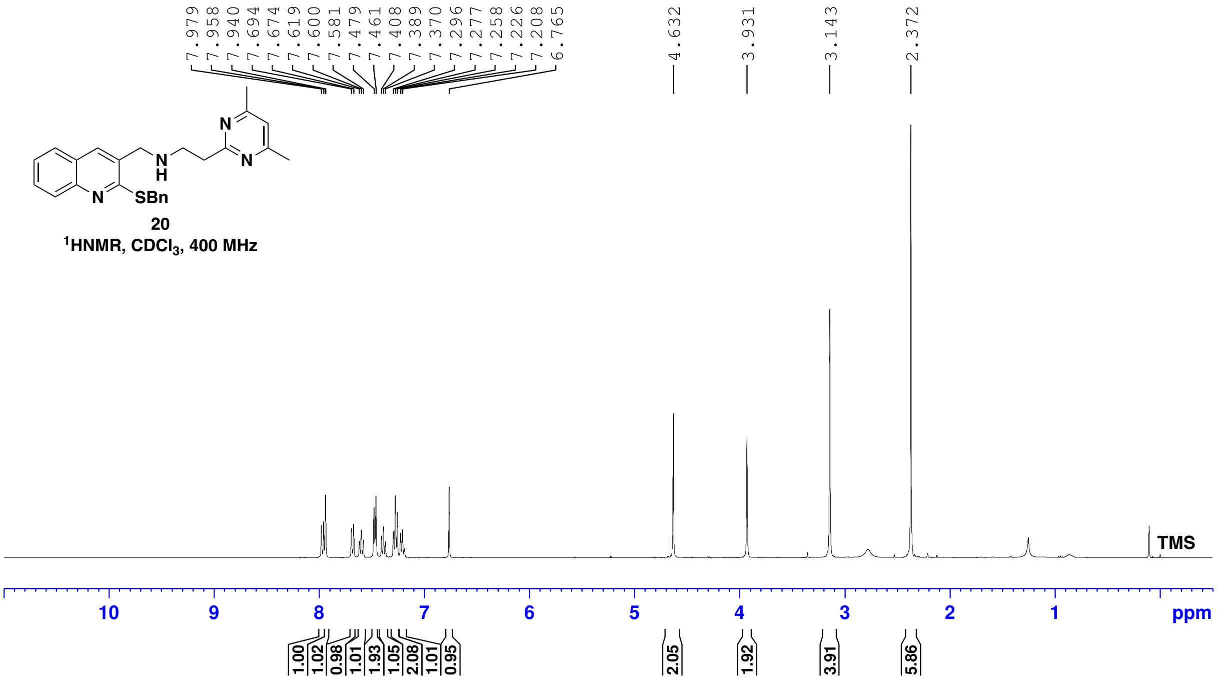
20
1HNMR, CDCl3, 400 MHz
7.979 7.958 7.940 7.694 7.674 7.619 7.600 7.581 7.479 7.461 7.408 7.389 7.370 7.296 7.277 7.258 7.226 7.208 6.765
4.632
3.931
3.143
2.372
TMS
1.00 1.02 0.98 1.01 1.93 1.05 2.08 1.01 0.95
2.05
1.92
3.91
5.86
10 9 8 7 6 5 4 3 2 1 ppm

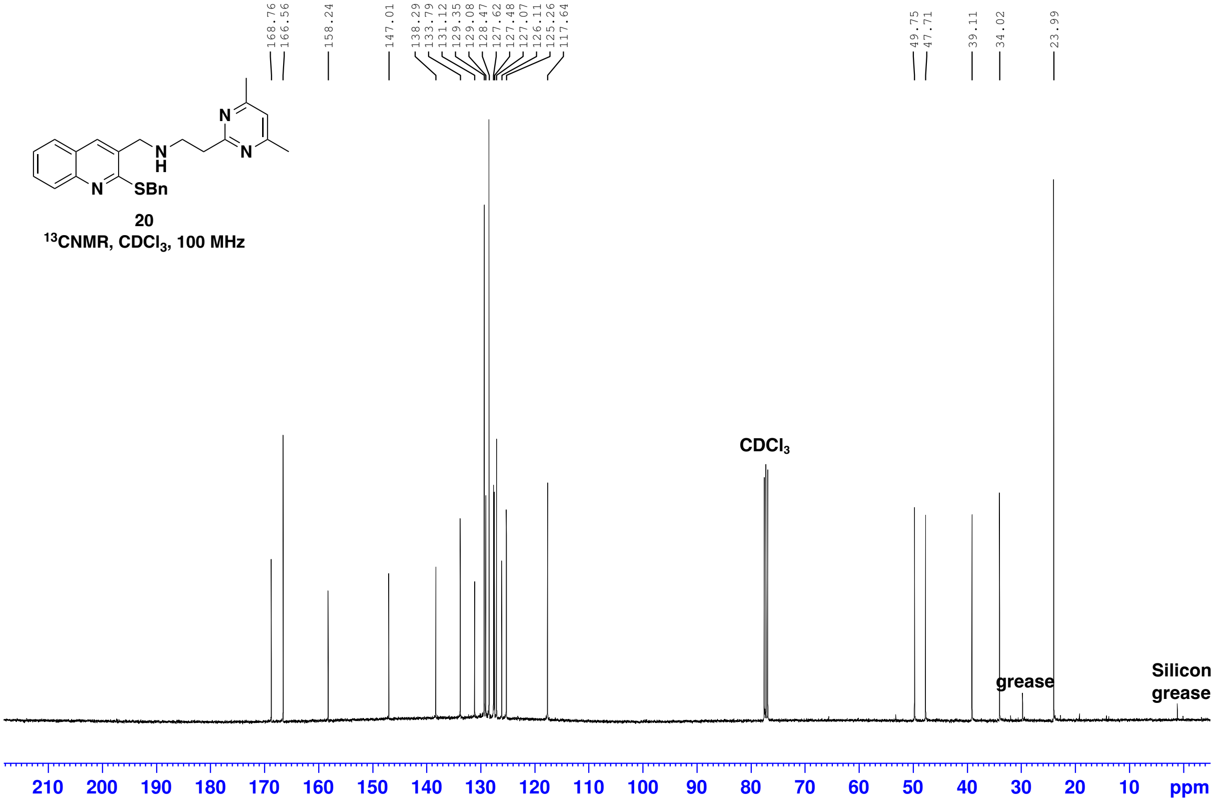
20
13CNMR, CDCl3, 100 MHz
168.76 166.56
158.24
147.01
138.29 133.79 131.12 129.35 129.08 128.47 127.62 127.48 127.07 126.11 125.26 117.64
49.75 47.71
39.11
34.02
23.99
CDCl3
grease
Silicon grease
210 200 190 180 170 160 150 140 130 120 110 100 90 80 70 60 50 40 30 20 10 ppm

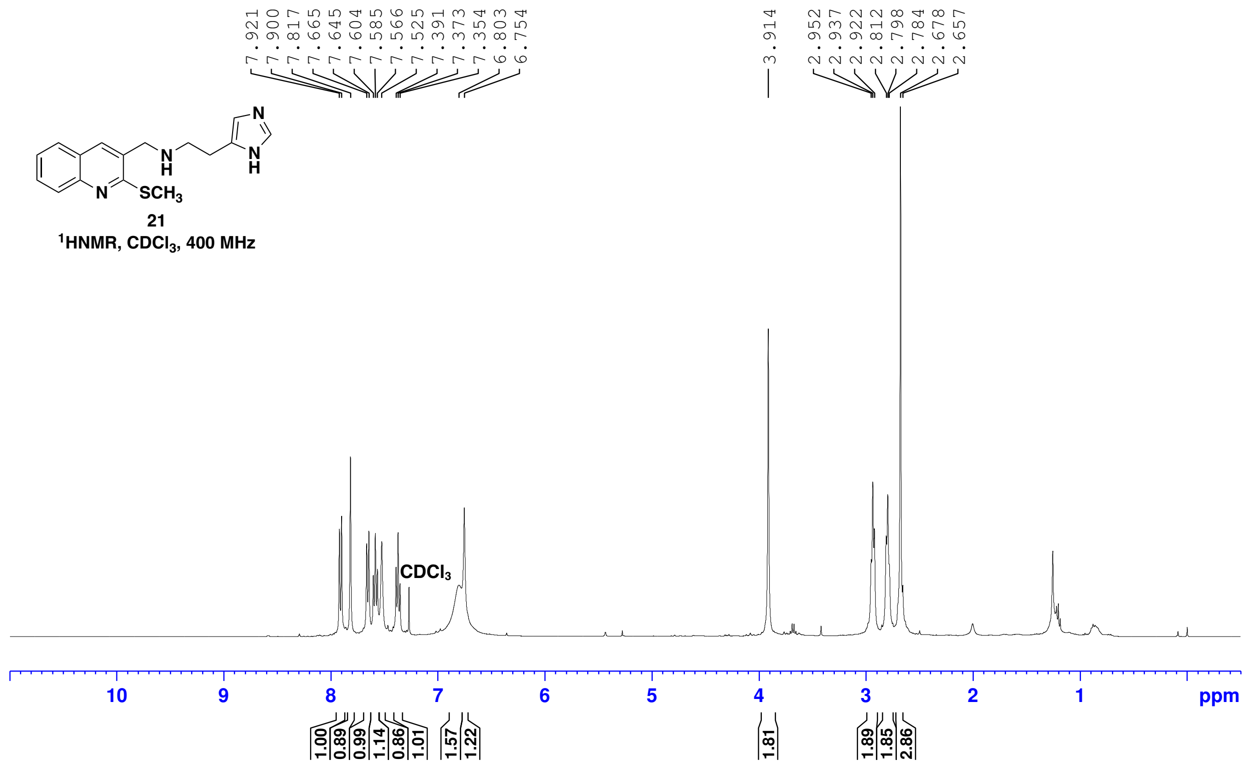
7.921 7.900 7.817 7.665 7.645 7.604 7.585 7.566 7.525 7.391 7.373 7.354 6.803 6.754
3.914
2.952 2.937 2.922 2.812 2.798 2.784 2.678 2.657
N
N
H
H
N
SCH3
21
1HNMR, CDCl3, 400 MHz
CDCl3
10 9 8 7 6 5 4 3 2 1 ppm
1.00 0.89 0.99 1.14 0.86 1.01 1.57 1.22
1.81
1.89 1.85 2.86

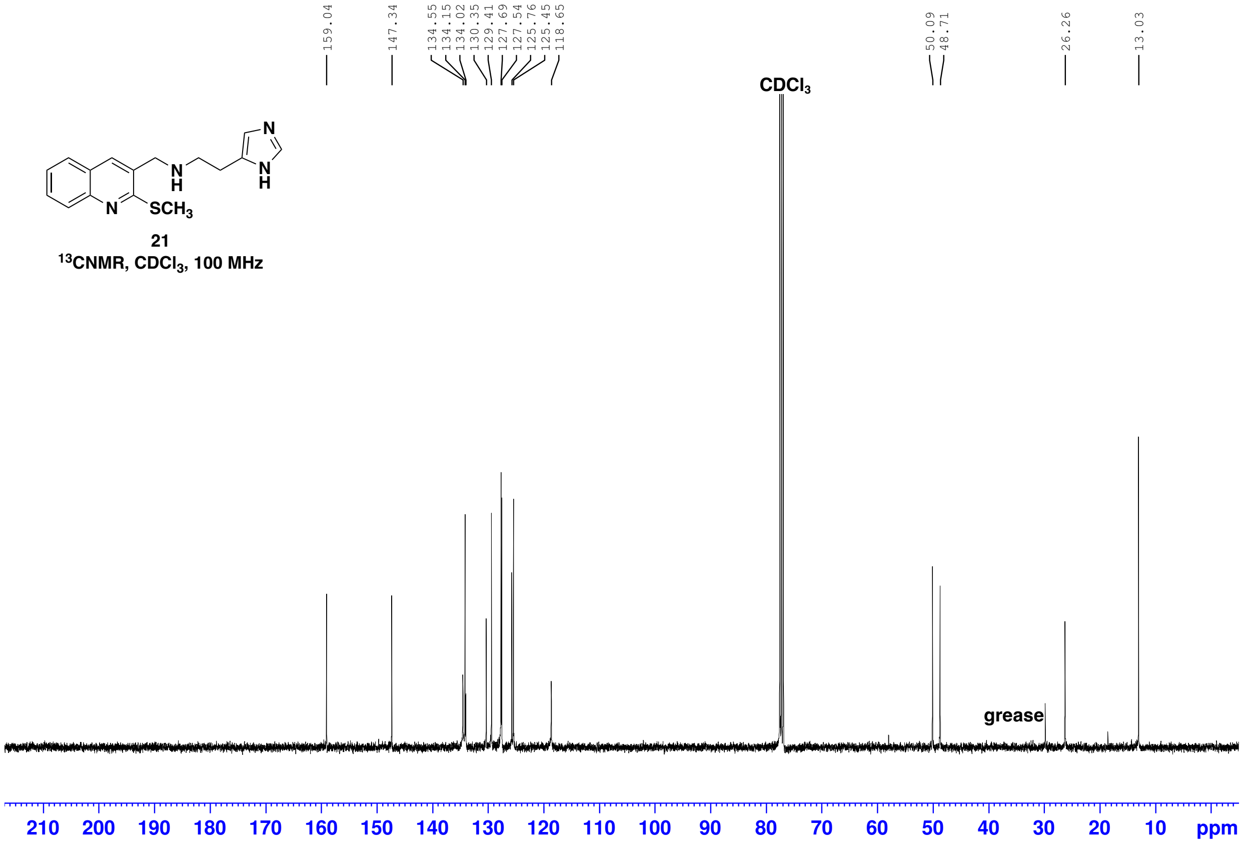
159.04
147.34
134.55 134.15 134.02 130.35 129.41 127.69 127.54 125.76 125.45 118.65
50.09 48.71
26.26
13.03
CDCl3
N
N
H
H
N
SCH3
21
13CNMR, CDCl3, 100 MHz
grease
210 200 190 180 170 160 150 140 130 120 110 100 90 80 70 60 50 40 30 20 10 ppm

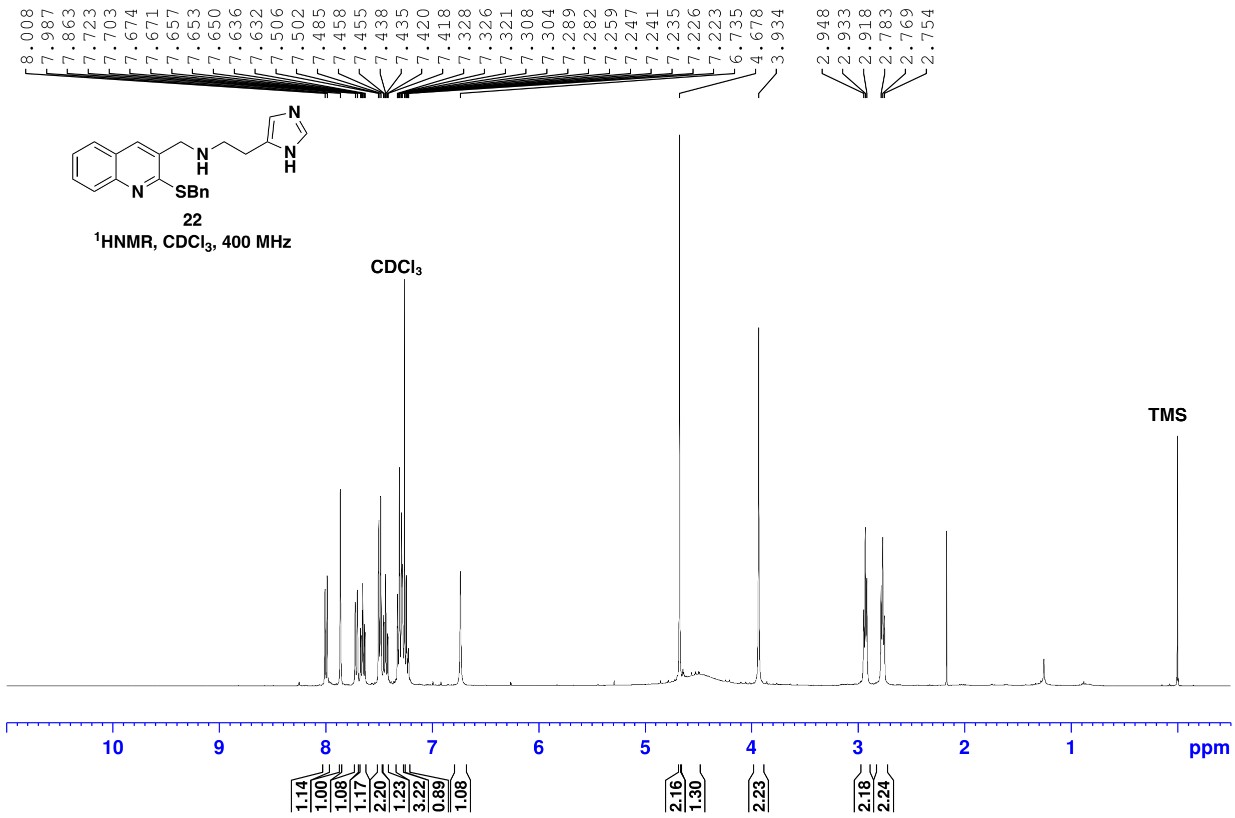
22
1HNMR, CDCl3, 400 MHz
CDCl3
TMS
ppm

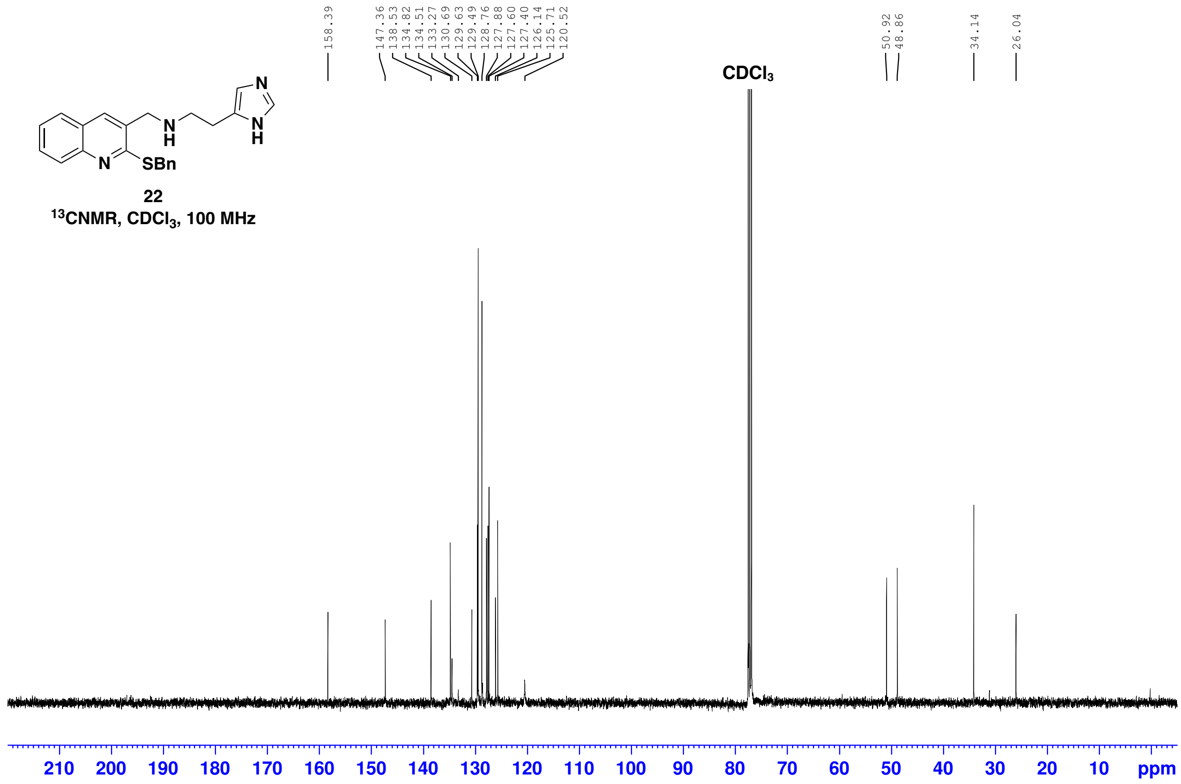
22
13CNMR, CDCl3, 100 MHz
CDCl3
ppm

**Table S1. Electron Transport Chain Controls for *E. coli* DK8 pASH20 and PA Inverted Membrane Vesicles.**

| Compound | % PA ETC Inhibition at 32 μg/mL or 4 μg/mL, or $IC_{50}$ in μg/mL | % EC ETC Inhibition at 4 μg/mL or $IC_{50}$ in μg/mL |
|---|---|---|
| **5** | 54% (32 μg/mL) | 9% (4 μg/mL) |
| **6** | 44 | 3% (4 μg/mL) |
| **7** | 72% (32 μg/mL) | 6% (4 μg/mL) |
| **8** | 26 | 0% (4 μg/mL) |
| **9** | 74% (32 μg/mL) | 22% (4 μg/mL) |
| **10** | 57 | 14% (4 μg/mL) |
| **11** | 20% (4 μg/mL) | 4% (4 μg/mL) |
| **12** | ND | ND |
| **13** | 74% (32 μg/mL) | 20% (4 μg/mL) |
| **14** | 26 | 16% (4 μg/mL) |
| **15** | 8.3 | 6% (4 μg/mL) |
| **16** | 14 | 9% (4 μg/mL) |
| **17** | 65% (32 μg/mL) | 0% (4 μg/mL) |
| **18** | 62% (32 μg/mL) | 5%(4 μg/mL) |
| **19** | 20% | 1.3% (4 μg/mL) |
| **20** | 67% (32 μg/mL) | 17% (4 μg/mL) |
| **21** | 39 | 5% (4 μg/mL) |
| **22** | 15 | 14 |

**ND** – compound 12 was insoluble at concentrations > 16 μg/mL but displayed some PA and EC ETC inhibition.

**Table S2. Physical Properties for 1 and 2 and 5-22**

| Compound | MW (g/mol) | clogP | globularity | RB | PBF | PTSA (Å) |
|---|---|---|---|---|---|---|
| **1** | 352 | 3.35 | 0.095 | 7 | 1.16 | 27.63 |
| **2** | 427 | 4.92 | 0.077 | 9 | 1.033 | 27.63 |
| **5** | 289 | 3.02 | 0.074 | 7 | 0.854 | 27.63 |
| **6** | 365 | 4.59 | 0.108 | 9 | 1.043 | 27.63 |
| **7** | 303 | 2.74 | 0.032 | 8 | 0.633 | 27.63 |
| **8** | 379 | 4.31 | 0.038 | 10 | 0.741 | 27.63 |
| **9** | 317 | 3.27 | 0.047 | 9 | 0.808 | 27.63 |
| **10** | 393 | 4.84 | 0.096 | 11 | 1.178 | 27.63 |
| **11** | 337 | 3.68 | 0.05 | 6 | 0.712 | 27.63 |
| **12** | 413 | 5.25 | 0.041 | 8 | 0.845 | 27.63 |
| **13** | 343 | 4.09 | 0.033 | 7 | 0.668 | 27.63 |
| **14** | 419 | 5.66 | 0.032 | 9 | 0.786 | 27.63 |
| **15** | 383 | 4.77 | 0.046 | 7 | 0.904 | 27.63 |
| **16** | 459 | 6.51 | 0.05 | 9 | 0.942 | 27.63 |
| **17** | 311 | 3.53 | 0.026 | 6 | 0.571 | 27.63 |
| **18** | 387 | 5.09 | 0.065 | 8 | 0.816 | 27.63 |
| **19** | 338 | 2.46 | 0.128 | 6 | 0.974 | 49.11 |
| **20** | 414 | 4.03 | 0.035 | 8 | 0.642 | 49.11 |
| **21** | 298 | 1.33 | 0.061 | 6 | 0.623 | 48.78 |
| **22** | 374 | 2.9 | 0.098 | 8 | 1.004 | 48.78 |

Calculated on ChemDraw 22.2 and entry-way.org[15]
